# Occurrence, distribution, and ecological risk assessment of heavy metals in Chao Phraya River, Thailand

**DOI:** 10.1038/s41598-024-59133-0

**Published:** 2024-04-10

**Authors:** Sarima Niampradit, Nuttapohn Kiangkoo, Rachaneekorn Mingkhwan, Wissanupong Kliengchuay, Suwalee Worakhunpiset, Yanin Limpananont, Surat Hongsibsong, Duangrat Inthorn, Kraichat Tantrakarnapa

**Affiliations:** 1 Department of Social and Environmental Medicine, Faculty of Tropical Medicine, Mahidol University, Krung Thep Maha Nakhon, 10400 Thailand; 2 Environment, Health & Social Impact Unit, Faculty of Tropical Medicine, Mahidol University, Krung Thep Maha Nakhon, Thailand; 3 Research Institute for Health Sciences, Chiang Mai University, Chiang Mai, Thailand; 4 Department of Environmental Health Sciences, Faculty of Public Health, Mahidol University, Krung Thep Maha Nakhon, Thailand; 5 Center of Excellence on Environmental Health and Toxicity (EHT), Krung Thep Maha Nakhon, Thailand

**Keywords:** Heavy metals, Heavy metal pollution index, Ecological risk assessment, Species sensitivity distribution (SSD), Chao Phraya River, Environmental sciences, Endocrinology, Risk factors, Environmental chemistry, Ecological modelling

## Abstract

Understanding heavy metals in rivers is crucial, as their presence and distribution impact water quality, ecosystem health, and human well-being. This study examined the presence and levels of nine heavy metals (Cd, Cr, Cu, Fe, Hg, Mn, Ni, Pb, and Zn) in 16 surface water samples along the Chao Phraya River, identifying Fe, Mn, Zn, and Cr as predominant metals. Although average concentrations in both rainy and dry seasons generally adhered to WHO guidelines, Mn exceeded these limits yet remained within Thailand’s acceptable standards. Seasonal variations were observed in the Chao Phraya River, and Spearman’s correlation coefficient analysis established significant associations between season and concentrations of heavy metals. The water quality index (WQI) demonstrated varied water quality statuses at each sampling point along the Chao Phraya River, indicating poor conditions during the rainy season, further deteriorating to very poor conditions in the dry season. The hazard potential index (HPI) was employed to assess heavy metal contamination, revealing that during the dry season in the estuary area, the HPI value exceeded the critical threshold index, indicating the presence of heavy metal pollution in the water and unsuitable for consumption. Using the species sensitivity distribution model, an ecological risk assessment ranked the heavy metals’ HC5 values as Pb > Zn > Cr > Cu > Hg > Cd > Ni, identifying nickel as the most detrimental and lead as the least toxic. Despite Cr and Zn showing a moderate risk, and Cu and Ni posing a high risk to aquatic organisms, the main contributors to ecological risk were identified as Cu, Ni, and Zn, suggesting a significant potential ecological risk in the Chao Phraya River’s surface water. The results of this study provide fundamental insights that can direct future actions in preventing and managing heavy metal pollution in the river ecosystem.

## Introduction

Environmental pollutants, heavy metals are widely recognized for their toxicity, long atmospheric lifespan, and capacity for human body accumulation through bioaccumulation, such as cadmium (Cd), copper (Cu), chromium (Cr), iron (Fe), mercury (Hg), lead (Pb), nickel (Ni), manganese (Mn), and zinc (Zn)^1^. These metals are widely used in industrial and agricultural activities, and this trend continues to increase to support the rapid growth of the population^2^. The increased use of heavy metals leads to increased contamination in the environment, which has become an ecological and global public health concern. Heavy metals are highly soluble in the aquatic environments and therefore they can be absorbed easily by living organisms^3^. The presence of heavy metal contaminants in surface water gives rise to a host of toxic effects on aquatic organisms, eliciting adverse outcomes for both the affected organisms and human health. These pollutants are absorbed directly from the water source as well as indirectly through the intricate food chains in aquatic ecosystems. Fishes and aquatic invertebrates bear the brunt of heavy metal toxicity, experiencing hindered developmental growth, heightened occurrences of developmental anomalies, compromised survival rates, particularly during the initial exogenous feeding phase, and even the risk of entire species extinction. Additionally, human health is endangered as heavy metals infiltrate the food chain, amplifying concerns associated with heavy metal contamination in surface water^4,5^. Human exposure to heavy metals can lead to both acute and chronic adverse health effects. Some heavy metals, including Arsenic (As), Cd, Pb, Hg and Uranium (U), have been appropriately explored to provide insight into their impact on mammalian reproductive systems^6^. Additionally, heavy metals such as Cd, Zn, Pb, Mn, Hg, and As exhibit properties of Endocrine-Disrupting Chemicals (EDCs) and have the potential to disrupt the endocrine system, leading to alterations in physiological functions^7^.

The sources of heavy metal contamination in the aquatic environment come from both natural and anthropogenic sources. Regarding natural sources, metals are formed through geographical processes such as the weathering of rocks containing metals^8^. Anthropogenic activities, including agricultural practices such as fertilizer, pesticide, and herbicide usage, discharge from factories and mining operations, and wastewater discharge from communities, also contribute to heavy metal contamination. Consequently, the presence of heavy metals has significantly increased due to human activities^9^. Monitoring studies in Thailand have revealed heavy metal contamination in diverse environments such as coastal areas, farmland, wetlands, mining areas, industrial zones, rivers, and estuaries^10^. The extensive impact is attributed to over 30,000 industrial establishments in the Chao Phraya River basin, contributing to river water pollution with harmful substances, including heavy metals discharged from industrial effluents^11^, This widespread environmental impact is observed across various ecosystems. Additionally, the Chao Phraya River, passing through densely populated Bangkok, is at risk of heavy metal pollution from both road traffic and water transport activities. Another study in Beijing, China, investigated urban stormwater runoff, emphasizing the elevated concentrations of toxic metals and rare earth elements^12,13^. The findings highlight the significant impact of vehicular activities, atmospheric deposition, and coal burning on runoff pollution.

The Chao Phraya River is an important river in Thailand. It is formed by the confluence of two main tributaries from the northern region, namely the Ping River and the Nan River, which converge at Pak Nam Pho in the Mueang Nakhon Sawan District of Nakhon Sawan Province. The Chao Phraya River supports more than 13 million people in 11 provinces and is used for various activities, including water supply, irrigation, water resource utilization in agriculture and manufacturing sectors, as well as water transportation^14,15^. The Marine Department of Thailand’s report in 2020 revealed that the water quality of the Chao Phraya River fails to meet the established standards, primarily due to anthropogenic activities and the discharge of wastewater from neighboring industries and communities^16^. A previous study reported a situation of heavy metal pollution in the Chao Phraya River estuary, indicating significant accumulation of Cd, Cu, Cr, and Pb in the water near the river mouth. It was also noted that Thai people may be ingesting Hg and Pb through fish from this river estuary^17^. Nevertheless, there is a limited number of studies about the heavy metals contamination status of the Chao Phraya River, which is a concern for many studies focusing on human health and the ecological impact caused by micropollutant runoff from various sectors, such as agriculture, municipal sources, industries, and residential areas^18^. However, the previous studies showed on heavy metal contamination did not comprehensively assessed the potential ecological risks linked to these metals. As a result, it is crucial to undertake more investigations to explore the ecological consequences that may arise from contamination by heavy metals.

The objectives of this study were (1) to explore the occurrence and distribution of heavy metals in the surface water of the Chao Phraya River, (2) to analyze the correlation between physicochemical parameters and the distribution patterns of these identified heavy metals, (3) to investigate the seasonal variations in heavy metal concentrations, (4) to assess the ecological risk arising from heavy metal contamination in the river’s surface water. The results of this study provide substantial scientific data that can contribute to water quality management and addressed heavy metal pollution for future policies development.

## Materials and methods

### Study area

**Figure 1:**
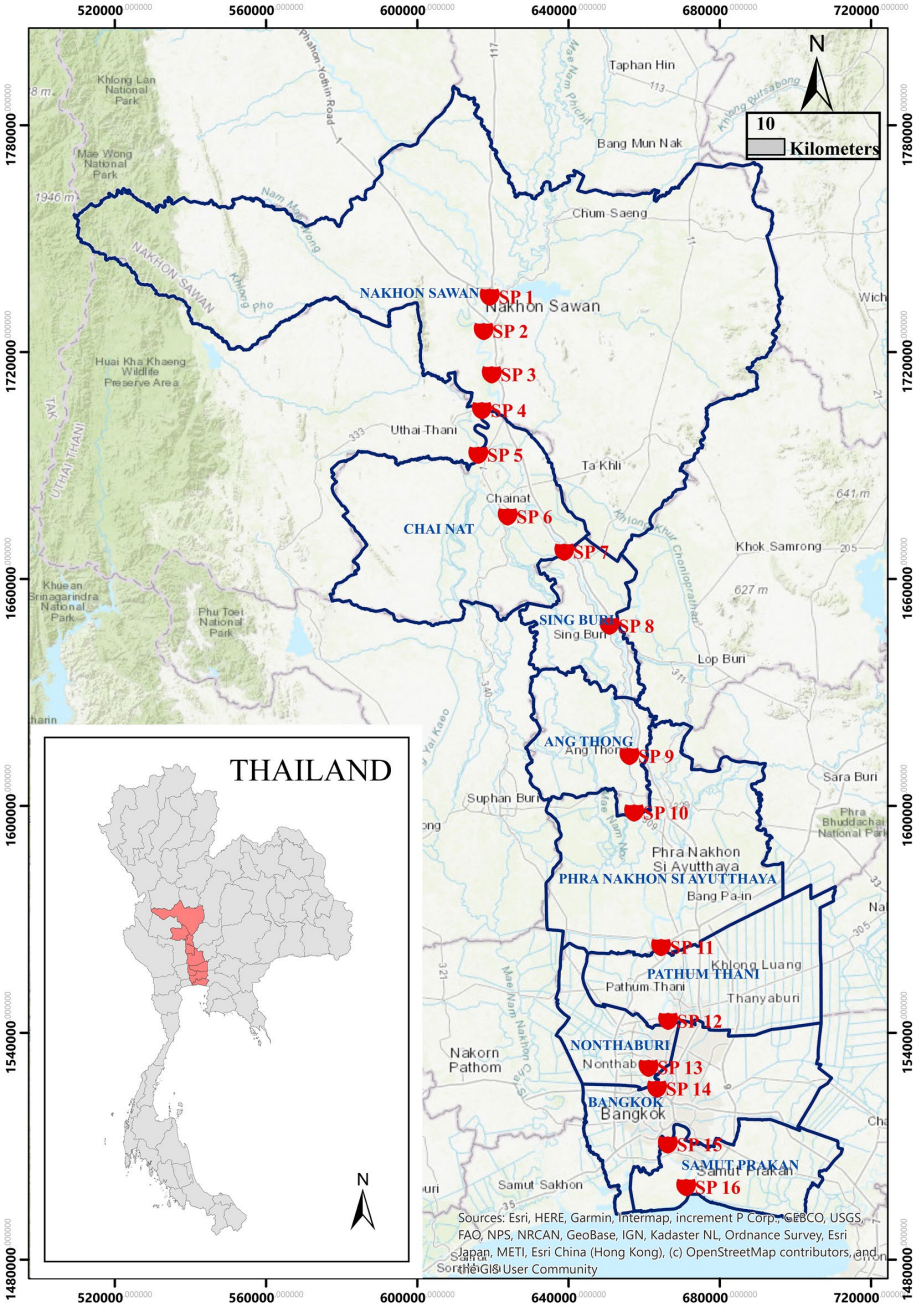
Study location and sampling site along Chao Phraya River, Thailand (ArcGIS version10.4, ESRI Education Site License Program, Thailand).

The Chao Phraya River basin, located centrally in Thailand, spans an extensive land area of 20,523.42 km^2^. Originating from the confluence of the Ping and Nan Rivers in Nakhon Sawan province, the primary river stretches for 372 km. Its course takes it southwards, passing through Uthai Thani, Chainat, Sing Buri, Ang Thong, Phra Nakhon Si Ayutthaya, Pathum Thani, Nonthaburi, and Bangkok, before ultimately emptying into the Gulf of Thailand at Pak Nam, Samut Prakan Province. The climate in the Chao Phraya Basin is primarily influenced by the southwest monsoon and northeast monsoon, resulting in three distinct seasons: the rainy season (May–October), winter (November–mid-February), and summer (mid-February–early May), with occasional influence from Depression Storms. The annual average temperature is 28.5 °C, reaching its lowest point in December-January and peaking in April. The Chao Phraya River Basin receives an average rainfall of 1231.0 mm, with September experiencing the highest monthly rainfall at an average of 241.7 mm. This study involved the collection of surface water samples from 16 designated sampling points (SP) situated along the Chao Phraya River during both the rainy season (July 2022) and the dry season (December 2022). Figure 1 illustrates a map that encompasses all the sampling points, while detailed descriptions of each sampling point can be found in Table S1.

### Water sampling and analysis

The water samples (100 ml) were collected at 1 m of the river depth in polyethylene bottles using a Kemmerer water sampler and added with 200 µl of concentrated nitric acid (HNO_3_; with a purity of 65% supplied by Merck, Darmstadt, Germany) for metal preservation and then stored in an icebox at 4 °C before transfer to the laboratory. The physicochemical parameters (pH, dissolved oxygen, and conductivity) were measured using HACH HQ40d multiparameter meter (HACH Company, Colorado, USA). The preparation and analysis methods were followed US EPA procedure^19^. For sample preparation, acid-preserved samples were filtered into polypropylene centrifuge tube by using Whatman Ashless Filter Paper (Grade 41, 110 mm) and then added HNO_3_ to adjust the acid concentration of samples to approximate 1% (v/v) nitric acid solution. The analysis of eight heavy metals were performed using an atomic absorption spectrometry (model ZA3000 Series, (Hitachi High Technologies, Tokyo, Japan). For Hg, it was analyzed by Mercury Analyzer (Model MA-3000, Nippon Instruments Corporation, Tokyo, Japan). Instrumental operating conditions for atomic absorption spectrometry in heavy metal analysis are shown in the Supplementary Material (Table S2).

### Statistical analysis

The statistical analysis was conducted using the XLSTAT software (Data Analysis and Statistical Solution for Microsoft Excel, Addinsoft, Paris, France 2017) to calculate the descriptive statistics such as mean, max, and min of heavy metals concentration in water samples. There are 2 steps of statistical analysis (i) the initial step involved the normality test by using Shapiro–Wilk, followed by (ii) test of concentration difference by using the application of the Mann–Whitney U test. These tests were employed to detect significant variances (with a significance level of p < 0.05) in the concentrations of heavy metals found in surface water samples, both across different study locations and throughout various seasons. Spearman’s correlation coefficient was used to assess the relationship between heavy metal and physicochemical parameters. The levels of heavy metals in different seasons were analyzed using the Mann–Whitney U Test, while Ecological risk assessment of heavy metals were performed using RStudio software in a package of “SSDforR” of SSD curves.

### Quality assurance and quality control

All the quality control and assurance procedures were validated following USEPA guidance. Laboratory consumables and solvents used in the experiments were guaranteed analytical grades or higher and routinely checked for possible contamination. In order to mitigate the risk of interference and cross-contamination, a thorough cleansing process was undertaken on the equipment and glassware. This involved immersing them in diluted HNO_3_ solution for at least 24 h prior to analysis and kept all containers and filters separate at each step for every sample. The supplementary data (Table S3) validates the accuracy of the calibration curve, with an R^2^ value exceeding 0.995, indicating satisfactory calibration. The %recovery for the external standard fell within the acceptable range, ranging from 88.5 to 112.32%. Additionally, the limit of quantification (LOQ) and limit of detection (LOD) were provided. Certified reference material (*Environ*MAT Groundwater, High (ES-H)) was employed during sample analysis to ensure quality control in the research, with instrument readings taken in triplicate for each sample.

### Water quality index (WQI)

The WQI is a tool used to assess the status of water quality, and the calculation method employed in this study is the Weighted Arithmetic Index method developed by Ref.^20^. For this study, the selected parameters include pH, DO, EC, Cr, Cu, Mn, Ni, Pb, and Zn. The calculation formula for the WQI follows Eq. (1)

1 $$\mathrm{WQI }=\frac{\sum {\text{W}}_{\text{n}}{{\text{Q}}}_{\text{n}}}{\sum {\text{W}}_{n}},$$

where W_n_ is unit weight factor of each parameter calculated as Eq. (2), and Q_n_ represents the sub-index value, calculated according to Eq. (3)

2 $${{\text{W}}}_{{\text{n}}} =\frac{1}{\sum \frac{1}{{\text{S}}_{\text{n}}}}\bigg/{\text{S}}_{\text{n}},$$

where S_n_ is standard desirable value of nth, representing the permissible limit for water class II according to the Pollution Control Department of Thailand^21^. The sum of all selected parameters unit weight factors (W_n_) must equal 1 (unity).

3 $${{\text{Q}}}_{{\text{n}}} = \frac{\text{[}\left({\text{V}}_{\text{n}}- \, {\text{V}}_{0}\right)\text{]}}{\text{[(}{\text{S}}_{\text{n}}- \, {\text{V}}_{0}\text{)]}} \, \times {10}\text{,}$$

where V_n_ is the average concentration of nth parameters and V_0_ is actual values of the parameter in pure water. Generally, V_0_ is set to 0 for most parameters, except for pH, where it is equal to 7, and for DO, where it is set to 14. To interpret the results of the WQI, the water quality status is categorized into five classes: Excellent (0–25), Good (26–50), Poor (51–75), Very Poor (76–100), and Unsuitable for consumption (> 100), as shown in Table S4.

### Heavy metal pollution index (HPI)

Heavy metal pollution index (HPI) was employed to assess heavy metal pollution and overall water quality, as demonstrated in several previous studies^22–24^. The HPI method was developed by Venkata et al.^25^. It is a valuable tool for quantitatively evaluating the extent of heavy metal contamination in water sources. This method is used to determine whether the water sample is contaminated with heavy metal or not by assessing the level of overall heavy metal, which is calculated as shown in Eq. (4)

4 $$\mathrm{HPI }= \frac{\sum_{\text{i=1}}^{\text{n}}{\text{W}}{\text{i}}{{\text{Q}}}_{{\text{i}}}}{\sum_{\text{i=1}}^{\text{n}}{\text{W}}{\text{i}}}.$$

Within this study, the quantity of heavy metals is represented by the variable ‘n.’ To calculate the unit weight of a specific parameter, denoted as ‘W_i_,’ Eq. (5) is employed. Furthermore, the sub-index value of the ‘ith’ parameter, indicated as ‘Q_i_,’ can be obtained by following the calculation method illustrated in Eq. (6).

5 $${\text{W}}{\text{i}}\text{ } = \frac{\text{K}}{{S}_{i}},$$

where K is constant equal to 1, and S_i_ is the standard permissible limit value of the ith parameters (µg/l), which is refers to the highest permissible limit of any heavy metal prescribed by the selected organization. In this study, WHO guidelines for drinking water quality and Water quality standards by Pollution Control Department of Thailand were selected as data sources for each heavy metal^26,27^.

6 $${{\text{Q}}}_{{\text{i}}} =\sum_{\text{i = 1}}^{\text{n}}\frac{\text{(}{{\text{M}}}_{{\text{i}}} \, - \, {{\text{I}}}_{{\text{i}}}\text{) }}{\text{(}{{\text{S}}}_{{\text{i}}} \, - \, {{\text{I}}}_{{\text{i}}}\text{)}},$$

where Mi is the monitored value of the heavy metal of the ith parameters (µg/l), I_i_ is the ideal value of the ith parameters (µg/l), which refers to the lowest acceptable limit of heavy metals prescribed by the selected organization. Especially, it underscores the crucial role played by HPI values, wherein a value lower than 100 indicates the absence of heavy metal pollution and ensures water suitability for consumption. Conversely, if the HPI value surpasses 100, it indicates heavy metal contamination, implying potential adverse health consequences associated with consuming such water.

### Ecological risk assessment

The risk quotient (RQ) was selected to assess the ecological risks of heavy metal and calculated by the following equation (Eq. 7). The risk characterization can be divided into 3 levels: low risk (RQ < 0.1), medium risk (0.1 ≤ RQ < 1), and high risk (1 ≤ RQ).

7 $$\mathrm{RQ }=\frac{\text{MEC}}{{\text{PNEC}}},$$

where MEC is the measured environment concentration, PNEC is the predicted no-effect concentration which is calculated as HC_5_ divided by assessment factor (AF) as shown in Eq. (8)

8 $$\mathrm{PNEC }=\frac{{\text{HC}}_{5}}{\text{AF}},$$

where HC_5_ or 5% hazard concentration is the cumulative concentration when the proportion of harmful species on the Species Sensitivity Distributions (SSD) curve reaches 5%^28^. A lower HC_5_ value signifies a more potent heavy metal in terms of toxicity. This study employed the SSD model to integrate toxicity data obtained from the US EPA ECOTOX database^29^ and used 50% effective concentration (EC_50_) and 50% lethal concentration (LC_50_) as toxicity endpoint. For species selection, there are 3 groups of freshwater species native to Thailand, consist of algae, invertebrate (crustaceans and other invertebrates) and fish^30–32^. In the case of the same species, the geometric means were used for estimating the mean value as representative of the species. The list of heavy metal toxicity data obtained from ECOTOX database was shown in Supplementary data (Tables S5–S11). AF to derive a PNEC in SSD method in this study was set to dues to an evaluation of the uncertainties around the derivation of the 5th percentile^33^.

### Ethics approval and consent to participate

The study was approved by the Ethics Committee of Faculty of Tropical Medicine, Mahidol University TMEC 21-082 in compliance with Declaration of Helsinki, ICH guideline for Good Clinical Practice and other international Guidelines for Human Research Protection. Inform Consent is not applicable for this study due to the secondary data obtained.

## Results and discussion

### Occurrence and distribution of heavy metal

In this study, the presence and levels of nine heavy metals (Cd, Cr, Cu, Fe, Hg, Mn, Ni, Pb, and Zn) in the surface water of the Chao Phraya River were determined. The forms of metals are soluble in the water that we can examine the concentration. Heavy metals are highly soluble in aquatic environments and therefore they can be absorbed easily by living organisms. The various forms of metals in water can be detected namely; the Chromium (III) oxide chromium (III) hydroxide and Chromium (VI) oxide. For Nickel, in general it is water insoluble, however, it can be soluble in the form of nickel carbonate. Concerning the Copper, Copper (II) Sulfate is soluble in water, whereas the zinc can be soluble in water in the form of zinc ion, zine Chloride and zinc carbonate^34^.

The results of the analyzed heavy metals in both the rainy and dry seasons are presented in Table 1, and the concentrations of each heavy metal at each sampling point are presented in Tables S12 and S13. Cd and Hg were not detected in any samples. For rainy season, the average concentrations of heavy metals were in the order of Fe (566.91 µg/l) > Mn (158.47 µg/l) > Zn (10.03 µg/l) > Cr (3.02 µg/l) > Cu (2.36 µg/l) > Pb (2.33 µg/l) > Ni (1.33 µg/l), respectively. In contrast, during the dry season, the levels of heavy metals were in the order of Fe (1245.66 µg/l) > Mn (151.57 µg/l) > Zn (6.18 µg/l) > Cr (2.70 µg/l) > Cu (1.15 µg/l) > Ni (1.88 µg/l) > Pb (0.78 µg/l). Fe, Mn, Zn, and Cr were observed as predominant metals in the Chao Phraya River. Based on the drinking water quality guidelines, the average concentration of most heavy metals remained below the recommended values^27^, except for Mn, which exceeded the WHO guidelines (80 µg/l) but remained within the acceptable level of Thailand’s Water Quality Standard (1000 µg/l)^26^. In this study, most heavy metal concentrations increased in the estuary area of Samut Prakan province (SP15–16).

**Table 1:** Occurrence and distribution of heavy metal in surface water of Chao Phraya River.

Heavy metal	Season	Concentration of heavy metal (µg/L)	SD	CV (%)	Detection (%)	Distribution (%)	Thailand water quality standard (µg/l)^26^	WHO guidelines (µg/l)^27^
Cd	Rainy	< LOQ	–	–	0	–	5	3
	Dry	< LOQ	–	–	0	–		
Cr	Rainy	0.98–13.51	2.99	98.89	100	0.40	50	50
	Dry	< LOQ–15.47	4.83	178.62	100	0.19		
Cu	Rainy	1.81–5.63	0.89	37.81	100	0.32	100	2000
	Dry	< LOQ–5.62	1.93	52.13	87.5	0.26		
Fe	Rainy	154.63–1321.07	331.74	58.52	100	75.78	NA	NA
	Dry	107.17–5444.33	1350.60	108.43	100	88.19		
Hg	Rainy	< LOQ	–	–	0	–	2	6
	Dry	< LOQ	–	–	0	–		
Mn	Rainy	46.58–1018.07	233.77	147.52	100	21.18	1000	80
	Dry	53.91–486.67	111.46	73.54	100	10.73		
Ni	Rainy	< LOQ–1.73	0.24	18.04	100	0.18	100	70
	Dry	< LOQ–3.22	0.74	39.55	100	0.13		
Pb	Rainy	< LOQ–8.63	3.82	64.33	75	0.79	50	10
	Dry	< LOQ	–	–	12.5	0.06		
Zn	Rainy	5.33–17.10	3.41	34.02	100	1.34	1000	NA
	Dry	2.40–19.50	3.97	64.29	100	0.44		

*CV* coefficient of variation, *NA* not applicable.

*LOD value of each element were presented in Table S4.

The elevated concentration of heavy metals in estuaries, in comparison to other parts of the river, is attributed to the deposition and transportation of heavy metals in the environment, leading to their accumulation in estuaries through surface runoff and river flows. This accumulation is influenced by the dynamic interaction between freshwater and saltwater in estuarine environments, resulting in the buildup of pollutants in water^35,36^. Comparison of heavy metal concentrations with other researches in Thailand and other countries in Southeast Asia are listed in Table 2. Compared with previous studies in Thailand, the concentrations of Cr, Fe, Mn, and Pb monitored in the surface water of downstream Chao Phraya River during 2013 were lower than the sampling water in 2022^37^. For other rivers in Thailand, heavy metal concentration in this study was higher than Mun River^38^ but lower than Tha Chin River^39^. Compared to the heavy metals concentration in other regions in Southeast Asia, the concentration of most heavy metals detected in the Chao Phraya River were slightly higher than other Southeast Asia rivers due to intense industrial and communities nearby the river^40–41^. The increasing trend of heavy metal pollution in Southeast Asia countries may cause by unbalanced economic growth, essential environmentally friendly technology, and a lack of regional law enforcement^24^.

**Table 2:** Comparison of heavy metal (µg/L) in surface water of river in Thailand and other countries in southeast Asia.

Country	Location	Cd	Cr	Cu	Fe	Hg	Mn	Ni	Pb	Zn	References
Thailand	Chao Phraya River (n = 16)	< LOQ	< LOQ–15.47	< LOQ–5.63	107.17–5444.33	< LOQ	46.58–1018.07	< LOQ–3.22	< LOQ–8.63	2.41–19.5	This study
Thailand	Chao Phraya River (n = 20)	0.029–0.193	–	–	–	0.001–0.007	–	0.052–0.541	0.190–4.428	0.102–1.047	^46^
Thailand	Chao Phraya River (n = 9)	ND–0.32	0.43–6.49	1.12–14.22	ND–409.60	–	ND–1189.0	3.94–23.54	0.32–1.88	28.06–160.60	^37^
Thailand	Tha Chin River (n = 38)	ND–50	-	10–1200	–	–	–	–	ND–1040	160–7470	^39^
Thailand	Mun River (n = 104)	–	–	ND–2.51	8.04–536.05	–	0.03–527.00	–	–	ND–6.24	^38^
Cambodia	Tonle Sap-Bassac River (n = 11)	ND	0.10–0.46	0.25–1.62	ND–22.76	–	ND–4.15	ND–0.56	ND–0.17	ND	^37^
Indonesia	Citarum River (n = 10)	ND–0.06	ND–2.80	0.51–6.94	17.65–557.40	–	6.89–638.00	0.17–9.66	0.10–1.30	3.28–44.26	^37^
Indonesia	Winongo River (n = 8)	0–10	0–20	0–40	200–1680	–	–	–	40–690	–	^41^
Malaysia	Linggi River (n = 15)	0.01–2.61	–	0.06–3.06	6.83–179.66	–	2.59–40.69	0.11–0.39	0.03–0.27	1.16–6.35	^24^
Malaysia	Semenyih River (n = 8)	0.12–0.68	1.64–5.46	0.84–7.33	280.76–488.60	0–0.96	30.11–59.79	0.29–0.88	0.70–3.08	33.10–49.19	^40^
Viet Nam	Saigon River (n = 8)	0.20–254.00	0.01–8.98	0.55–16.51	7.59–2590.0	–	9.03–179.80	ND–3.90	ND–6.55	5.38–311.10	^37^

The water quality parameters of each sampling point are presented in the Supplementary Data (Table S1). The results of water quality in this study are similar to the water quality report of the main river in Thailand by the Thailand Marine Department^16^, which indicated that the dissolved oxygen (DO) levels in most of the sampling points were lower than the surface water quality standard (not less than 6 mg/l) set by the Pollution Control Department of Thailand^42^. The main potential sources of low water quality were municipal wastewater (70%), industrial wastewater (20%), and agricultural activities (10%)^43^.

For the normality test, it was found that almost all parameters were not normally distributed (p value < 0.05), except for the pH (p value = 0.3502), as presented in the Supplementary Material (Table S15). The analysis of seasonal variation in heavy metals involved the implementation of the Mann–Whitney U Test. Results revealed that Cr, Cu, Fe, Ni, Pb, and Zn exhibited significant changes in concentration throughout different seasons, while Mn did not show any significant change. There is a possibility that certain heavy metals may be higher during the dry season, while others are higher in the rainy season. According to the literature review, during the rainy season, the likelihood of higher concentrations of heavy metals arises from increased river levels due to rainfall and the runoff of heavy metal-containing materials into the river^44^. On the other hand, the probability of higher levels of heavy metals during the dry season is attributed to the lower river levels due to reduced water volume and flow, along with increased evaporation from water bodies, resulting in elevated levels of heavy metals^45^. In this study, higher concentrations of heavy metals were determined in the Chao Phraya River during the rainy season (Cr, Cu, Ni, Pb, and Zn) than in the dry season (Fe). This may conclude that heavy metal levels in the Chao Phraya River are more influenced by runoff during the rainy season. Spearman correlation analysis was conducted to examine the relationship between seasonal variation and heavy metal concentrations, along with physicochemical parameters (dissolved oxygen, pH, and electrical conductivity), in the surface water of the Chao Phraya River (Table 3). The results indicate significant correlations between several heavy metals and physiochemical parameters. Notably, electrical conductivity (EC) demonstrates a negative correlation with dissolved oxygen (DO) with the value of − 0.55 and a positive correlation with Mn (− 0.02), Ni (− 0.75), and Zn (− 0.35). Furthermore, Ni exhibits a strong negative correlation with DO (− 0.75), emphasizing the potential influence of seasonal variations on the relationship between DO and heavy metal concentrations in the Chao Phraya River. These findings contribute valuable insights into the complex dynamics of heavy metal distribution in relation to seasonal changes in the river’s water quality.

**Table 3:** Spearman correlation analysis of heavy metal and physicochemical parameters in surface water of Chao Phraya River.

Variables	DO	pH	EC	Cr	Cu	Fe	Mn	Ni	Pb	Zn
DO	1.00									
pH	0.17	1.00								
EC	− 0.55*	− 0.11	1.00							
Cr	− 0.09	0.36*	0.42*	1.00						
Cu	− 0.26	0.31	0.52*	0.86*	1.00					
Fe	0.01	0.05	0.06	0.18	0.09	1.00				
Mn	− 0.02	0.05	0.15	0.24	0.31	0.57*	1.00			
Ni	− 0.75*	− 0.21	0.52*	0.15	0.37*	0.17	0.18	1.00		
Pb	− 0.14	0.10	0.22	0.37*	0.34	0.19	0.32	0.07	1.00	
Zn	− 0.35	0.11	0.40*	0.70*	0.81*	− 0.06	0.20	0.41*	0.32	1.00

*Correlation is significant at the 0.05 level (2-tailed), According to Wuensch^47^ and Shajib et al.^12^ the range of absolute value of r is 0.00–0.19 “Very weak”; 0.20–0.39 “weak”; 0.40–0.59 “moderate”; 0.60–0.79 “strong”; 0.80–1.0 “very strong”^12,47^.

### Water quality index (WQI)

**Figure 2:**
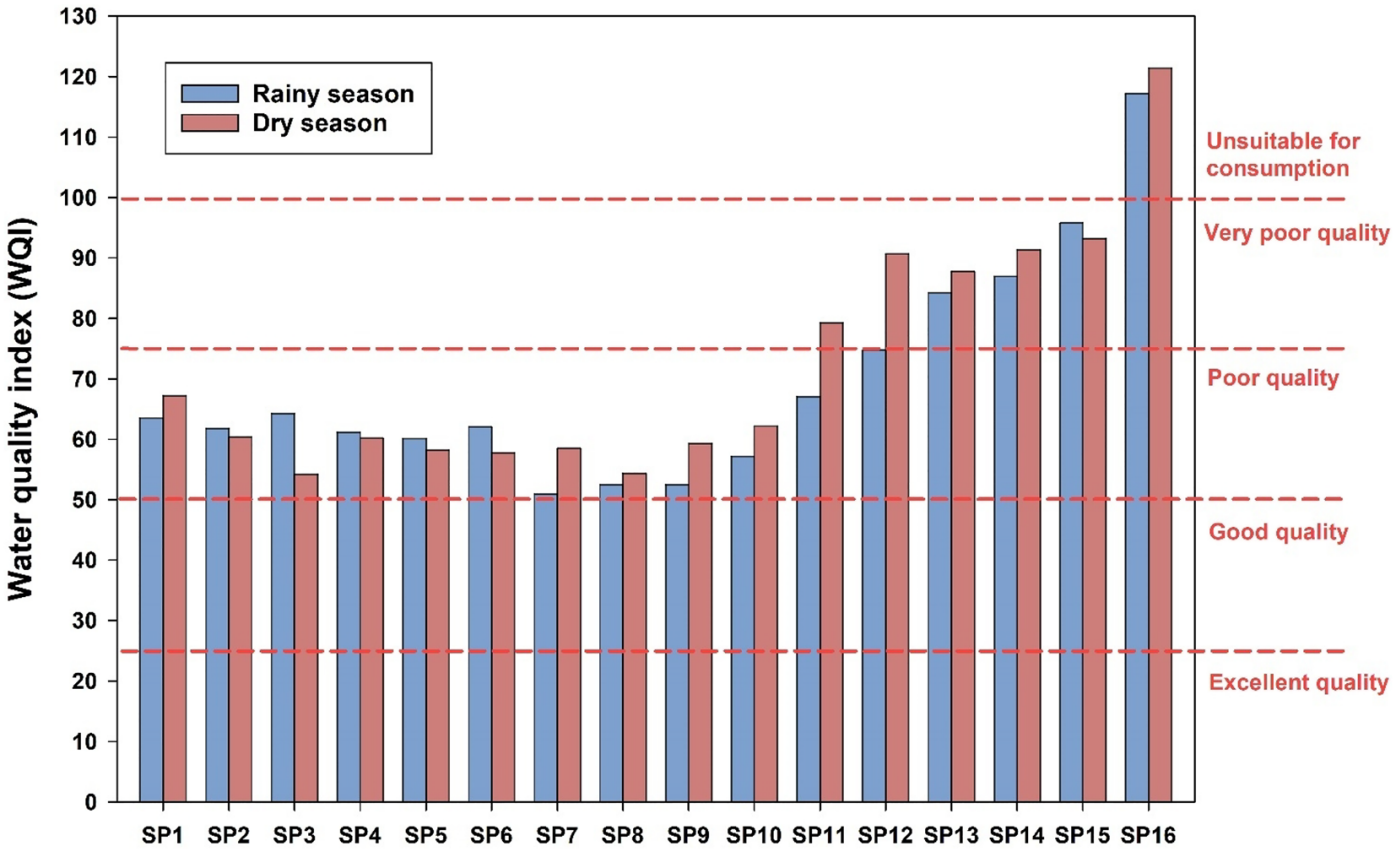
Water quality index (WQI) in surface water of Chao Phraya River.

The results of the water quality assessment are presented in Fig. 2 and Table S14. The WQI results revealed distinct water quality statuses at each sampling point. During the rainy season, the water quality was generally categorized as “Poor”, with some variations observed among the sampling points. However, during the dry season, the water quality deteriorated further, with several sampling points falling into the “Very Poor” category. Notably, at SP16, the WQI exceeded 100, indicating that the water is “Unsuitable for consumption”.

Comparing these findings with the Thailand State of Pollution Report 2022^48^, the water quality situation in the Chao Phraya River is reported as fair in the upper and middle parts of the river, calculated using the Unweighted Multiplicative River Water Index. However, the lower part of the river is reported to be in poor condition. Parameters such as DO, biochemical oxygen demand (BOD), total coliform bacteria (TCB), fecal coliform bacteria (FCB), and heavy metals were reported to exceed standard limits. The primary contributors to the deterioration in water quality are primarily municipal wastewater, agricultural runoff, aquaculture, and livestock activities without an adequate waste management system. These findings underscore the urgent need for comprehensive water management strategies to address the identified sources of pollution and enhance the overall water quality in the Chao Phraya River.

### Heavy metal pollution index (HPI)

**Figure 3:**
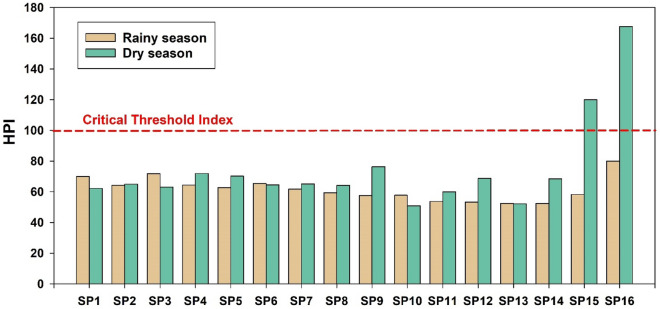
Heavy metal pollution index (HPI) in surface water of Chao Phraya River.

The HPI values for all sampling sites in the Chao Phraya River during both seasons are illustrated in Fig. 3. The highest HPI values were observed at SP15 (119.91) and SP16 (167.61) during the dry season, surpassing the critical threshold index of 100. This indicates that water in these areas is contaminated with heavy metals and unsuitable for consumption. Conversely, for the remaining sampling points, HPI values ranged from 52.31 to 71.81, with an average of 61.52 during the rainy season, and from 50.91 to 76.24, with an average of 64.44 during the dry season, all below the threshold index. These findings underscore the need for further investigation and remediation measures in areas exceeding the critical threshold to safeguard water quality and public health.

### Ecological risk assessment

The species sensitivity distribution curve of heavy metals, generated using the R program, is presented in supplementary data (Figs. S1–S7). The toxicity data used in this study were obtained from the ECOTOX database provided by US EPA. The HC_5_ values of the heavy metals were as follows: Pb (227.61 μg/l) > Zn (91.29 μg/l) > Cr (83.43 μg/l) > Cu (16.32 μg/l) > Hg (8.34 μg/l) > Cd (6.07 μg/l) > Ni (5.53 μg/l). HC_5_ values less than 20 μg/l were observed for Cd, Cu, Hg, and Ni, indicating them as the main factors contributing to ecological risk in the river. The smaller the HC_5_ value, the more sensitive the aquatic organisms are^49^. Therefore, Ni was identified as the most harmful metal to freshwater ecology, while Pb was found to be the least toxic. However, the SSD curve for Fe and Mn is not present due to limited toxicity data for these metals.

According to the risk quotient (RQ) results presented in Table 4, which includes RQ_max_ calculated based on the maximum concentration and RQ_mean_ calculated using the average concentration of each heavy metal, Cr exhibits low-risk levels during both seasons, with RQ values well below the threshold of 0.1, indicating minimal ecological concern. Cu and Zn present medium risk levels in both seasons. While Cu’s RQ values are relatively high, falling within the 0.1 to 1 range, Zn displays a moderate level of risk, suggesting potential ecological impacts that warrant attention and monitoring. Ni portrays high risk levels, especially during the dry season, with RQ values exceeding 1. This signifies a potential adverse impact on the aquatic ecosystem, necessitating immediate attention and intervention. In this study, Cu, Ni, and Zn were identified as the primary contributors to ecological risk in the water bodies of the Chao Phraya River. Copper, known for its high toxicity to aquatic organisms and ecosystems, exhibits algaecide properties leading to decreased algal growth when accidentally released into water bodies. Since algae form the foundation of food chains, their abundance directly affects the availability of food for a diverse range of aquatic animals^50,51^. Furthermore, Ni’s significant toxic potential in surface waters lies in its ability to accumulate in sediments and impact various levels of the food chain^52,53^. Zn, when present at toxic levels, adversely affects the physical structure and functioning of fish, leading to overall weakness, significant histological alterations in multiple organs, and impeded growth and maturation processes^54,55^. In summary, the findings reveal a substantial potential ecological risk in the surface water of the Chao Phraya River. However, the presence of these harmful metals can vary significantly among various aquatic organisms, depending on the species and prevailing environmental conditions in the water.

**Table 4:**
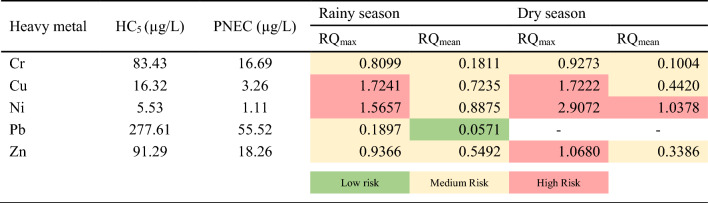
Risk quotient of heavy metals in Chao Phraya River.

## Conclusion

This study investigated the occurrence, distribution, and ecological risk of various heavy metals (Cd, Cr, Cu, Fe, Hg, Mn, Ni, Pb, and Zn) in the surface water of the Chao Phraya River in Thailand. The outcomes revealed that the concentration of Mn was 2 times higher than the limit set by WHO. While the other heavy metals remained within acceptable levels. The Spearman correlation analysis revealed significant associations between seasonal variation and heavy metal concentrations in the Chao Phraya River. The Water Quality Index revealed poor water quality during the rainy season, worsening to very poor in the dry season, notably exceeding 100 at estuary, indicating unsuitability for consumption. In addition, the WQI from SP11–SP16 are classified as poor quality that relate to the location, SP11–SP16 are the provinces with higher density population compared to the upper part of this river. The observed poor water quality, particularly at the estuary, raises concerns for the well-being of aquatic life and communities dependent on the river. The Heavy Metal Pollution Index at the estuary of the Chao Phraya River during the dry season surpassed the critical threshold index, indicating the presence of heavy metal pollution in the water. Specifically, elevated levels of Cu, Ni and Zn were identified as significant contributors to this ecological risk, may pose a threat to various flora and fauna in the river. In addition, the sea water intrusion to the estuary might be the factor influencing the metals contamination. In conclusion, this study emphasizes the urgent need for targeted environmental management strategies to mitigate potential adverse effects on aquatic ecosystems in the Chao Phraya River. The findings, providing a foundational understanding, can guide future measures, including implementing proactive strategies such as stricter regulations on industrial discharges, community awareness programs, and regular monitoring of water quality, all of which could play pivotal roles in preventing and controlling heavy metal pollution within the Chao Phraya River ecosystem, fostering its long-term health and sustainability.

### Supplementary Information

Supplementary Information.
