# Supplementary material for: Occurrence, distribution, and ecological risk assessment of heavy metals in Chao Phraya River, Thailand
Source: Sci Rep. 2024 Apr 10;14:8366. doi: 10.1038/s41598-024-59133-0 (PMC11006942; doi:10.1038/s41598-024-59133-0)
Supplement: Supplementary file 1 — Supplementary Information. [file 41598_2024_59133_MOESM1_ESM.docx]

**Supplementary data for Occurrence, distribution, and Ecological risk assessment of heavy metals in Chao Phraya River, Thailand**

Sarima Niampradit^1,2^, Nuttapohn Kiangkoo^1,2^, Rachaneekorn Mingkhwan^1,2^, Wissanupong Kliengchuay^1,2^, Suwalee worakhunpiset^1,2^, Yanin Limpananont^1,2^, Surat Hongsibsong^3^, Duangrat Inthorn^4,5^, Kraichat Tantrakarnapa^1,2*^

^1^Department of Social and Environmental Medicine, Faculty of Tropical Medicine, Mahidol University, Bangkok, Thailand

^2^Environment, Health & Social Impact Unit, Faculty of Tropical Medicine, Mahidol University, Bangkok, Thailand

^3^Research Institute for Health Sciences, Chiang Mai University, Thailand

^4^Department of Environmental Health Sciences, Faculty of Public Health, Mahidol University, Thailand

^5^Center of Excellence on Environmental Health and Toxicity (EHT), Bangkok 10400, Thailand

- **Table S1** The sampling locations and their physicochemical characteristics of water.
- **Instrumental Operating Conditions for Atomic Absorption Spectrometry in Heavy Metal Analysis**
- **Table S2** Instrumental operating conditions used for atomic absorption spectrometry.
- **Table S3** QA/QC parameter
- **Table S4** Water Quality Index (WQI) Classification
- **Table S5** Toxicity data of Cadmium (CAS number 7440439) in aquatic organism
- **Table S6** Toxicity data of Chromium (CAS number 7440473) in aquatic organism
- **Table S7** Toxicity data of Copper (CAS number 7440508) in aquatic organism
- **Table S8** Toxicity data of Mercury (CAS number 7439976) in aquatic organism
- **Table S9** Toxicity data of Nickel (CAS number 7440020) in aquatic organism
- **Table S10** Toxicity data of Lead (CAS number 7439921) in aquatic organism
- **Table S11** Toxicity data of Zinc (CAS number 7440666) in aquatic organism
- **Table S12** The concentration of heavy metals at each sampling point during the rainy season
- **Table S13** The concentration of heavy metals at each sampling point during the dry season
- **Table S14** The Water Quality Index (WQI) for each sampling point along the Chao Phraya River.
- **Table S15** The normality test by Shapiro-Wilk results for various water quality parameters
- **Fig S1** Species Sensitivity Distributions (SSD) curve of aquatic organisms for Cd
- **Fig S2** Species Sensitivity Distributions (SSD) curve of aquatic organisms for Cr
- **Fig S3** Species Sensitivity Distributions (SSD) curve of aquatic organisms for Cu
- **Fig S4** Species Sensitivity Distributions (SSD) curve of aquatic organisms for Hg
- **Fig S5** Species Sensitivity Distributions (SSD) curve of aquatic organisms for Ni
- **Fig S6** Species Sensitivity Distributions (SSD) curve of aquatic organisms for Pb
- **Fig S7** Species Sensitivity Distributions (SSD) curve of aquatic organisms for Zn

**Table S1**

The sampling locations and their physicochemical characteristics of water.

| Sample name | Location | DO (mg/L) | | pH | | Conductivity (μs/cm) | |
| --- | --- | --- | --- | --- | --- | --- | --- |
|  |  | Rainy season | Dry season | Rainy season | Dry season | Rainy season | Dry season |
| SP 1 | 15°41'11.1"N 100°06'42.9"E | 5.45 | 5.34 | 6.42 | 6.22 | 387 | 487 |
| SP 2 | 15°36'16.0"N 100°05'47.8"E | 5.25 | 5.36 | 6.60 | 6.61 | 393 | 363 |
| SP 3 | 15°30'02.6"N 100°06'57.2"E | 5.26 | 5.68 | 6.44 | 6.87 | 396 | 359 |
| SP 4 | 15°24'54.4"N 100°05'28.0"E | 5.13 | 5.00 | 6.67 | 6.76 | 388 | 358 |
| SP 5 | 15°18'31.4"N 100°04'53.9"E | 5.30 | 5.50 | 6.67 | 6.69 | 413 | 357 |
| SP 6 | 15°09'39.8"N 100°09'11.7"E | 5.02 | 5.60 | 6.66 | 6.68 | 417 | 368 |
| SP 7 | 15°04'33.4"N 100°17'31.4"E | 6.56 | 6.64 | 6.76 | 6.25 | 399 | 366 |
| SP 8 | 14°53'54.0"N 100°24'09.0"E | 6.35 | 7.25 | 6.74 | 6.27 | 395 | 368 |
| SP 9 | 14°35'11.7"N 100°26'57.4"E | 6.13 | 6.07 | 6.82 | 6.41 | 388 | 376 |
| SP 10 | 14°27'05.1"N 100°27'35.5"E | 5.67 | 5.25 | 6.70 | 6.54 | 369 | 380 |
| SP 11 | 14°07'46.6"N 100°31'26.1"E | 4.13 | 2.22 | 6.67 | 6.62 | 394 | 380 |
| SP 12 | 13°57'11.0"N 100°32'23.6"E | 2.95 | 1.13 | 6.63 | 6.33 | 368 | 401 |
| SP 13 | 13°50'28.5"N 100°29'28.9"E | 1.81 | 1.41 | 6.47 | 6.41 | 364 | 436 |
| SP 14 | 13°47'28.4"N 100°30'41.7"E | 1.45 | 0.91 | 6.46 | 6.43 | 453 | 674 |
| SP 15 | 13°39'28.0"N 100°32'14.5"E | 0.77 | 0.68 | 6.36 | 6.63 | 1365 | 1663 |
| SP 16 | 13°33'20.3"N 100°34'53.2"E | 1.73 | 1.89 | 6.73 | 6.93 | 10863 | 13767 |

**Instrumental Operating Conditions for Atomic Absorption Spectrometry in Heavy Metal Analysis**

The concentrations of heavy metals in all samples were determined using atomic absorption spectrometry (AAS). The instrument was adjusted according to the specifications detailed in Table S2, encompassing parameters such as wavelength, time constant, lamp current, photomultiplier tube (PMT) voltage, and the type of gas used.

**Table S2**

Instrumental operating conditions used for atomic absorption spectrometry.

| Heavy metal | Wavelength (nm) | Time Constant (s) | Lamp Current (mA) | PMT Voltage (V) | Gas |
| --- | --- | --- | --- | --- | --- |
| Cd | 228.8 | 0.2 | 12 | 240 | Ar |
| Cr | 359.3 | 0.1 | 10 | 230 | Ar |
| Cu | 324.8 | 0.5 | 8 | 220 | Ar |
| Fe | 248.3 | 0.1 | 12.5 | 450 | Ar |
| Mn | 279.5 | 0.1 | 9 | 310 | Ar |
| Ni | 232 | 0.5 | 12 | 430 | Ar |
| Pb | 283.3 | 0.5 | 12 | 260 | Ar |
| Zn | 307.6 | 0.1 | 10 | 300 | Ar |

**Table S3**

QA/QC parameter

| Heavy metal | Recovery (%) | LOD (µg/L) | LOQ (µg/L) | Regression coefficient (R^2^) |
| --- | --- | --- | --- | --- |
| Cd | 112.32 | 0.07 | 0.2 | 0.9999 |
| Cr | 110.71 | 0.16 | 0.5 | 0.9994 |
| Cu | 95.65 | 0.28 | 1.3 | 0.9980 |
| Fe | 102.26 | 0.3 | 3.4 | 0.9995 |
| Hg | 88.50 | 0.08 | 0.15 | 0.9988 |
| Mn | 107.35 | 0.09 | 0.2 | 1.0000 |
| Ni | 108.21 | 0.25 | 1 | 0.9994 |
| Pb | 100.22 | 1.01 | 3 | 0.9995 |
| Zn | 102.88 | 0.003 | 0.03 | 0.9979 |

**Table S4**

Water Quality Index (WQI) Classification

| Water quality index (WQI) | Water quality status |
| --- | --- |
| 0 – 25 | Excellent quality |
| 26 – 50 | Good quality |
| 51 – 75 | Poor quality |
| 76 – 100 | Very poor quality |
| > 100 | Unsuitable for consumption |

**Table S5**

Toxicity data of Cadmium (CAS number 7440439) in aquatic organism

| Species Scientific Name | Effect | Endpoint | Toxic concentration (µg/L) |
| --- | --- | --- | --- |
| Brachionus calyciflorus | Reproduction | EC50 | 70 |
| Brachionus calyciflorus | Mortality | LC50 | 1300 |
| Carassius auratus | Mortality | LC50 | 49 |
| Carassius auratus | Mortality | LC50 | 49 |
| Carassius auratus | Mortality | LC50 | 170 |
| Carassius auratus | Mortality | LC50 | 170 |
| Channa punctata | Mortality | LC50 | 18500 |
| Channa punctata | Mortality | LC50 | 34640 |
| Channa striata | Mortality | LC50 | 9497 |
| Channa striata | Mortality | LC50 | 14600 |
| Channa striata | Mortality | LC50 | 16290 |
| Channa striata | Mortality | LC50 | 19400 |
| Channa striata | Mortality | LC50 | 19490 |
| Channa striata | Mortality | LC50 | 19540 |
| Chlamydomonas reinhardtii | Population | EC50 | 1 |
| Chlamydomonas reinhardtii | Population | EC50 | 2 |
| Chlamydomonas reinhardtii | Biochemistry | EC50 | 2 |
| Chlamydomonas reinhardtii | Biochemistry | EC50 | 4 |
| Chlamydomonas reinhardtii | Population | EC50 | 350 |
| Chlamydomonas reinhardtii | Population | EC50 | 440 |
| Chlamydomonas reinhardtii | Population | EC50 | 670 |
| Chlamydomonas reinhardtii | Behavior | EC50 | 899 |
| Chlamydomonas reinhardtii | Behavior | EC50 | 2248 |
| Chlamydomonas reinhardtii | Biochemistry | EC50 | 9600 |
| Chlamydomonas reinhardtii | Biochemistry | EC50 | 14000 |
| Chlamydomonas reinhardtii | Biochemistry | EC50 | 37300 |
| Chlamydomonas reinhardtii | Biochemistry | EC50 | 69400 |
| Cyprinus carpio | Mortality | LC50 | 2 |
| Cyprinus carpio | Mortality | LC50 | 5 |
| Cyprinus carpio | Mortality | LC50 | 7 |
| Cyprinus carpio | Mortality | LC50 | 240 |
| Cyprinus carpio | Mortality | LC50 | 300 |
| Cyprinus carpio | Mortality | LC50 | 450 |
| Cyprinus carpio | Mortality | LC50 | 3000 |
| Cyprinus carpio | Mortality | LC50 | 4260 |
| Cyprinus carpio | Mortality | LC50 | 17050 |
| Cyprinus carpio | Mortality | LC50 | 21070 |
| Cyprinus carpio | Mortality | LC50 | 165000 |
| Daphnia lumholtzi | Mortality | LC50 | 83 |
| Daphnia lumholtzi | Mortality | LC50 | 619 |
| Daphnia lumholtzi | Mortality | LC50 | 1585 |
| Daphnia lumholtzi | Mortality | LC50 | 2325 |
| Gambusia affinis | Mortality | LC50 | 18510 |
| Gambusia affinis | Cell(s) | EC50 | 21350 |
| Hypophthalmichthys molitrix | Mortality | LC50 | 43000 |
| Lemna minor | Behavior | EC50 | 60 |
| Lemna minor | Population | EC50 | 90 |
| Lemna minor | Population | EC50 | 100 |
| Lemna minor | Population | EC50 | 191 |
| Lemna minor | Growth | EC50 | 200 |
| Lemna minor | Population | EC50 | 360 |
| Lemna minor | Population | EC50 | 470 |
| Lemna minor | Population | EC50 | 650 |
| Lemna minor | Population | EC50 | 1600 |
| Lemna trisulca | Population | EC50 | 26 |
| Lemna trisulca | Population | EC50 | 76 |
| Scenedesmus quadricauda | Population | EC50 | 8 |
| Simocephalus vetulus | Mortality | LC50 | 24 |

**Table S6**

Toxicity data of Chromium (CAS number 7440473) in aquatic organism

| Species Scientific Name | Effect | Endpoint | Toxic concentration (µg/L) |
| --- | --- | --- | --- |
| Brachionus calyciflorus | Reproduction | EC50 | 2900 |
| Brachionus calyciflorus | Mortality | LC50 | 8300 |
| Carassius auratus | Mortality | LC50 | 660 |
| Chlorella pyrenoidosa | Physiology | EC50 | 5000 |
| Cyprinus carpio | Mortality | LC50 | 14300 |
| Cyprinus carpio | Mortality | LC50 | 18400 |
| Cyprinus carpio | Mortality | LC50 | 21200 |
| Cyprinus carpio | Mortality | LC50 | 93600 |
| Lecane hamata | Mortality | LC50 | 4410 |
| Lecane hamata | Enzyme(s) | EC50 | 5690 |
| Lecane luna | Enzyme(s) | EC50 | 1180 |
| Lecane luna | Enzyme(s) | EC50 | 1920 |
| Lecane luna | Mortality | LC50 | 3260 |
| Lecane quadridentata | Enzyme(s) | EC50 | 1650 |
| Lecane quadridentata | Enzyme(s) | EC50 | 1880 |
| Lecane quadridentata | Mortality | LC50 | 4500 |
| Lemna minor | Population | EC50 | 3899.7 |
| Lemna minor | Growth | EC50 | 8500 |
| Lemna minor | Growth | EC50 | 35000 |
| Nitzschia palea | Physiology | EC50 | 800 |
| Oreochromis mossambicus | Mortality | LC50 | 170000 |
| Oreochromis mossambicus | Mortality | LC50 | 179000 |
| Oreochromis mossambicus | Mortality | LC50 | 217500 |
| Scenedesmus quadricauda | Population | EC50 | 190 |
| Scenedesmus quadricauda | Population | EC50 | 540 |
| Simocephalus vetulus | Mortality | LC50 | 50 |

**Table S7**

Toxicity data of Copper (CAS number 7440508) in aquatic organism

| Species Scientific Name | Effect | Endpoint | Toxic concentration (µg/L) |
| --- | --- | --- | --- |
| Brachionus calyciflorus | Behavior | EC50 | 14 |
| Brachionus calyciflorus | Behavior | EC50 | 15 |
| Brachionus calyciflorus | Behavior | EC50 | 16 |
| Brachionus calyciflorus | Mortality | LC50 | 18 |
| Brachionus calyciflorus | Mortality | LC50 | 25 |
| Brachionus calyciflorus | Reproduction | EC50 | 26 |
| Brachionus calyciflorus | Mortality | LC50 | 26 |
| Brachionus calyciflorus | Mortality | LC50 | 31 |
| Brachionus calyciflorus | Mortality | LC50 | 31 |
| Brachionus calyciflorus | Behavior | EC50 | 37 |
| Brachionus calyciflorus | Behavior | EC50 | 67 |
| Brachionus calyciflorus | Behavior | EC50 | 92 |
| Brachionus calyciflorus | Behavior | EC50 | 220 |
| Brachionus calyciflorus | Behavior | EC50 | 228 |
| Brachionus plicatilis | Mortality | LC50 | 63 |
| Channa punctata | Mortality | LC50 | 339 |
| Channa punctata | Mortality | LC50 | 342 |
| Channa punctata | Mortality | LC50 | 365 |
| Channa punctata | Mortality | LC50 | 372 |
| Channa punctata | Mortality | LC50 | 387 |
| Channa punctata | Mortality | LC50 | 395 |
| Channa punctata | Mortality | LC50 | 432 |
| Channa punctata | Mortality | LC50 | 477 |
| Channa striata | Mortality | LC50 | 5192 |
| Channa striata | Mortality | LC50 | 9550 |
| Channa striata | Mortality | LC50 | 11270 |
| Channa striata | Mortality | LC50 | 12400 |
| Channa striata | Mortality | LC50 | 12440 |
| Chlamydomonas reinhardtii | Population | EC50 | 0.38 |
| Chlamydomonas reinhardtii | Biochemistry | EC50 | 0.82 |
| Chlamydomonas reinhardtii | Biochemistry | EC50 | 0.90 |
| Chlamydomonas reinhardtii | Population | EC50 | 1.3 |
| Chlamydomonas reinhardtii | Biochemistry | EC50 | 3.1 |
| Chlamydomonas reinhardtii | Biochemistry | EC50 | 3.2 |
| Chlamydomonas reinhardtii | Physiology | EC50 | 953.2 |
| Chlorella saccharophila | Physiology | EC50 | 983 |
| Chlorella vulgaris | Population | EC50 | 550 |
| Cirrhinus mrigala | Mortality | LC50 | 90 |
| Cirrhinus mrigala | Mortality | LC50 | 110 |
| Cirrhinus mrigala | Mortality | LC50 | 120 |
| Cirrhinus mrigala | Mortality | LC50 | 140 |
| Cirrhinus mrigala | Mortality | LC50 | 150 |
| Cyprinus carpio | Mortality | LC50 | 50 |
| Cyprinus carpio | Mortality | LC50 | 200 |
| Cyprinus carpio | Mortality | LC50 | 300 |
| Cyprinus carpio | Mortality | LC50 | 800 |
| Cyprinus carpio | Mortality | LC50 | 1000 |
| Cyprinus carpio | Mortality | LC50 | 1200 |
| Cyprinus carpio | Mortality | LC50 | 1900 |
| Cyprinus carpio | Mortality | LC50 | 5000 |
| Daphnia lumholtzi | Mortality | LC50 | 9.4 |
| Daphnia lumholtzi | Mortality | LC50 | 54.6 |
| Daphnia lumholtzi | Mortality | LC50 | 67.2 |
| Daphnia lumholtzi | Mortality | LC50 | 83 |
| Fragilaria capucina | Population | EC50 | 21 |
| Gambusia affinis | Mortality | LC50 | 49 |
| Gambusia affinis | Mortality | LC50 | 56 |
| Gambusia affinis | Cell(s) | EC50 | 190 |
| Gambusia affinis | Mortality | LC50 | 250 |
| Gambusia affinis | Mortality | LC50 | 900 |
| Gambusia affinis | Mortality | LC50 | 1400 |
| Gambusia affinis | Mortality | LC50 | 2000 |
| Gambusia affinis | Mortality | LC50 | 2500 |
| Gambusia affinis | Mortality | LC50 | 2900 |
| Gambusia affinis | Mortality | LC50 | 3500 |
| Gambusia affinis | Mortality | LC50 | 5000 |
| Gambusia affinis | Mortality | LC50 | 6000 |
| Ictalurus punctatus | Mortality | LC50 | 51 |
| Ictalurus punctatus | Mortality | LC50 | 65 |
| Ictalurus punctatus | Mortality | LC50 | 1362 |
| Ictalurus punctatus | Mortality | LC50 | 1503 |
| Ictalurus punctatus | Mortality | LC50 | 1603 |
| Ictalurus punctatus | Mortality | LC50 | 1657 |
| Ictalurus punctatus | Mortality | LC50 | 1878 |
| Ictalurus punctatus | Mortality | LC50 | 2436 |
| Labeo rohita | Mortality | LC50 | 234 |
| Lecane luna | Enzyme(s) | EC50 | 0.002 |
| Lecane luna | Mortality | LC50 | 60 |
| Lecane luna | Enzyme(s) | EC50 | 620 |
| Lecane quadridentata | Enzyme(s) | EC50 | 1 |
| Lecane quadridentata | Enzyme(s) | EC50 | 16.8 |
| Lecane quadridentata | Mortality | LC50 | 330 |
| Lemna minor | Population | EC50 | 82.6098 |
| Lemna minor | Behavior | EC50 | 130 |
| Lemna minor | Population | EC50 | 130 |
| Lemna minor | Population | EC50 | 140 |
| Lemna minor | Population | EC50 | 320 |
| Lemna minor | Growth | EC50 | 1100 |
| Lemna minor | Population | EC50 | 1152 |
| Lemna minor | Population | EC50 | 1254 |
| Lemna minor | Population | EC50 | 1361 |
| Lemna minor | Population | EC50 | 1558 |
| Lemna trisulca | Population | EC50 | 228.8 |
| Lemna trisulca | Population | EC50 | 1398 |
| Lemna trisulca | Population | EC50 | 3495 |
| Lemna trisulca | Population | EC50 | 1310000 |
| Lemna trisulca | Population | EC50 | 1570000 |
| Lemna trisulca | Population | EC50 | 1640000 |
| Macrobrachium lamarrei | Mortality | LC50 | 65 |
| Oreochromis mossambicus | Mortality | LC50 | 110 |
| Oreochromis mossambicus | Mortality | LC50 | 150 |
| Oreochromis mossambicus | Mortality | LC50 | 230 |
| Oreochromis mossambicus | Mortality | LC50 | 310 |
| Oreochromis mossambicus | Mortality | LC50 | 440 |
| Oreochromis mossambicus | Mortality | LC50 | 590 |
| Oreochromis mossambicus | Mortality | LC50 | 1500 |
| Oreochromis mossambicus | Mortality | LC50 | 2800 |
| Oreochromis mossambicus | Mortality | LC50 | 4270 |
| Oreochromis niloticus | Mortality | LC50 | 600 |
| Oreochromis niloticus | Mortality | LC50 | 680 |
| Oreochromis niloticus | Mortality | LC50 | 1000 |
| Oreochromis niloticus | Mortality | LC50 | 1819 |
| Oreochromis niloticus | Mortality | LC50 | 58300 |
| Oreochromis niloticus | Mortality | LC50 | 63920 |
| Oreochromis niloticus | Mortality | LC50 | 73400 |
| Oscillatoria sp. | Physiology | EC50 | 1868.3 |
| Phormidium sp. | Population | EC50 | 6.4 |
| Scenedesmus dimorphus | Mortality | LC50 | 61.7 |
| Scenedesmus dimorphus | Mortality | LC50 | 62.3 |
| Scenedesmus dimorphus | Mortality | LC50 | 62.7 |
| Scenedesmus dimorphus | Mortality | LC50 | 76.9 |
| Scenedesmus dimorphus | Mortality | LC50 | 91.4 |
| Scenedesmus dimorphus | Mortality | LC50 | 91.8 |
| Selenastrum capricornutum | Population | EC50 | 61 |
| Simocephalus vetulus | Mortality | LC50 | 57 |
| Synedra ulna | Physiology | EC50 | 681.8 |

**Table S8**

Toxicity data of Mercury (CAS number 7439976) in aquatic organism

| Species Scientific Name | Effect | Endpoint | Toxic concentration (µg/L) |
| --- | --- | --- | --- |
| Brachionus calyciflorus | Mortality | LC50 | 60 |
| Brachionus plicatilis | Mortality | LC50 | 61 |
| Brachionus plicatilis | Mortality | LC50 | 610 |
| Carassius auratus | Mortality | LC50 | 0.7 |
| Carassius auratus | Mortality | LC50 | 120 |
| Caridina rajadhari | Mortality | LC50 | 4.8 |
| Caridina rajadhari | Mortality | LC50 | 5.8 |
| Caridina rajadhari | Mortality | LC50 | 6.9 |
| Caridina rajadhari | Mortality | LC50 | 9.1 |
| Channa striata | Mortality | LC50 | 1571 |
| Channa striata | Mortality | LC50 | 3787 |
| Channa striata | Mortality | LC50 | 4602 |
| Channa striata | Mortality | LC50 | 6148 |
| Channa striata | Mortality | LC50 | 6355 |
| Channa striata | Mortality | LC50 | 6400 |
| Cyprinus carpio | Mortality | LC50 | 160 |
| Cyprinus carpio | Mortality | LC50 | 180 |
| Cyprinus carpio | Mortality | LC50 | 210 |
| Cyprinus carpio | Mortality | LC50 | 330 |
| Cyprinus carpio | Mortality | LC50 | 500 |
| Cyprinus carpio | Mortality | LC50 | 570 |
| Cyprinus carpio | Mortality | LC50 | 620 |
| Cyprinus carpio | Mortality | LC50 | 710 |
| Cyprinus carpio | Mortality | LC50 | 770 |
| Cyprinus carpio | Mortality | LC50 | 940 |
| Heteropneustes fossilis | Mortality | LC50 | 99 |
| Labeo rohita | Mortality | LC50 | 16.3 |
| Labeo rohita | Mortality | LC50 | 16.8 |
| Labeo rohita | Mortality | LC50 | 17.2 |
| Labeo rohita | Mortality | LC50 | 17.8 |
| Labeo rohita | Mortality | LC50 | 18.3 |
| Labeo rohita | Mortality | LC50 | 18.6 |
| Labeo rohita | Mortality | LC50 | 19.1 |
| Labeo rohita | Mortality | LC50 | 19.7 |
| Labeo rohita | Mortality | LC50 | 20.7 |
| Labeo rohita | Mortality | LC50 | 21.1 |
| Labeo rohita | Mortality | LC50 | 21.5 |
| Labeo rohita | Mortality | LC50 | 21.7 |
| Oreochromis niloticus | Mortality | LC50 | 3710 |
| Oreochromis niloticus | Mortality | LC50 | 3800 |
| Oreochromis niloticus | Mortality | LC50 | 3920 |
| Poecilia reticulata | Mortality | LC50 | 200 |
| Scenedesmus quadricauda | Population | EC50 | 240 |
| Selenastrum capricornutum | Population | EC50 | 303 |

**Table S9**

Toxicity data of Nickel (CAS number 7440020) in aquatic organism

| Species Scientific Name | Effect | Endpoint | Toxic concentration (µg/L) |
| --- | --- | --- | --- |
| Brachionus calyciflorus | Mortality | LC50 | 4000 |
| Cyprinus carpio | Mortality | LC50 | 1300 |
| Cyprinus carpio | Mortality | LC50 | 1540 |
| Cyprinus carpio | Mortality | LC50 | 1640 |
| Cyprinus carpio | Mortality | LC50 | 2300 |
| Cyprinus carpio | Mortality | LC50 | 10400 |
| Cyprinus carpio | Mortality | LC50 | 28900 |
| Cyprinus carpio | Mortality | LC50 | 38300 |
| Gambusia affinis | Cell(s) | EC50 | 11800 |
| Gambusia affinis | Mortality | LC50 | 13590 |
| Gambusia affinis | Mortality | LC50 | 68000 |
| Gambusia affinis | Mortality | LC50 | 100000 |
| Gambusia affinis | Mortality | LC50 | 150000 |
| Gambusia affinis | Mortality | LC50 | 185000 |
| Gambusia affinis | Mortality | LC50 | 230000 |
| Gambusia affinis | Mortality | LC50 | 270000 |
| Gambusia affinis | Mortality | LC50 | 310000 |
| Gambusia affinis | Mortality | LC50 | 350000 |
| Gammarus sp. | Mortality | LC50 | 13000 |
| Gammarus sp. | Mortality | LC50 | 15200 |
| Scenedesmus acutus | Population | EC50 | 28.8 |
| Scenedesmus acutus | Physiology | EC50 | 36.4 |
| Scenedesmus acutus | Physiology | EC50 | 277.0 |
| Scenedesmus acutus | Physiology | EC50 | 315.2 |
| Scenedesmus acutus | Population | EC50 | 586.9 |
| Scenedesmus acutus | Physiology | EC50 | 669.1 |
| Scenedesmus acutus | Population | EC50 | 809.9 |
| Scenedesmus acutus | Population | EC50 | 1273.6 |
| Scenedesmus acutus | Physiology | EC50 | 2042.4 |
| Scenedesmus acutus | Physiology | EC50 | 6690.7 |
| Scenedesmus acutus | Physiology | EC50 | 8099.2 |
| Scenedesmus acutus | Physiology | EC50 | 16257.1 |
| Scenedesmus acutus | Physiology | EC50 | 18135.2 |

**Table S10**

Toxicity data of Lead (CAS number 7439921) in aquatic organism

| Species Scientific Name | Effect | Endpoint | Toxic concentration (µg/L) |
| --- | --- | --- | --- |
| Carassius auratus | Mortality | LC50 | 1660 |
| Channa striata | Mortality | LC50 | 39510 |
| Channa striata | Mortality | LC50 | 47310 |
| Channa striata | Mortality | LC50 | 50660 |
| Channa striata | Mortality | LC50 | 58400 |
| Channa striata | Mortality | LC50 | 58460 |
| Channa striata | Mortality | LC50 | 58540 |
| Chlamydomonas reinhardtii | Phototactic response | EC50 | 455.8 |
| Chlamydomonas reinhardtii | Phototactic response | EC50 | 1118.9 |
| Chlamydomonas reinhardtii | Phototactic response | EC50 | 1471.1 |
| Hydrilla verticillata | Chlorophyll | EC50 | 18648 |
| Lemna minor | Growth, general | EC50 | 8000 |
| Macrobrachium malcolmsonii | Mortality | LC50 | 9800 |
| Macrobrachium malcolmsonii | Mortality | LC50 | 91510 |
| Oreochromis niloticus | Mortality | LC50 | 2731 |
| Scenedesmus acuminatus | Population | EC50 | 250 |
| Scenedesmus quadricauda | Population | EC50 | 12160 |
| Simocephalus vetulus | Mortality | LC50 | 4500 |

**Table S11**

Toxicity data of Zinc (CAS number 7440666) in aquatic organism

| Species Scientific Name | Effect | Endpoint | Toxic concentration (µg/L) |
| --- | --- | --- | --- |
| Brachionus calyciflorus | Mortality | LC50 | 1300 |
| Chlamydomonas reinhardtii | Behavior | EC50 | 248.4 |
| Chlamydomonas reinhardtii | Behavior | EC50 | 2026.8 |
| Chlamydomonas reinhardtii | Behavior | EC50 | 2942.1 |
| Cyprinus carpio | Mortality | LC50 | 450 |
| Cyprinus carpio | Mortality | LC50 | 1340 |
| Cyprinus carpio | Mortality | LC50 | 1640 |
| Cyprinus carpio | Mortality | LC50 | 2250 |
| Cyprinus carpio | Mortality | LC50 | 7800 |
| Cyprinus carpio | Mortality | LC50 | 9200 |
| Cyprinus carpio | Mortality | LC50 | 14400 |
| Cyprinus carpio | Mortality | LC50 | 17000 |
| Cyprinus carpio | Mortality | LC50 | 23000 |
| Cyprinus carpio | Mortality | LC50 | 30000 |
| Daphnia lumholtzi | Mortality | LC50 | 437.5 |
| Daphnia lumholtzi | Mortality | LC50 | 2290 |
| Daphnia lumholtzi | Mortality | LC50 | 6704 |
| Gambusia affinis | Mortality | LC50 | 116 |
| Gambusia affinis | Cell(s) | EC50 | 52480 |
| Gambusia affinis | Mortality | LC50 | 53280 |
| Gambusia affinis | Mortality | LC50 | 90000 |
| Gambusia affinis | Mortality | LC50 | 100000 |
| Gambusia affinis | Mortality | LC50 | 115000 |
| Gambusia affinis | Mortality | LC50 | 120000 |
| Gambusia affinis | Mortality | LC50 | 140000 |
| Gambusia affinis | Mortality | LC50 | 150000 |
| Gambusia affinis | Mortality | LC50 | 50000 |
| Gambusia affinis | Mortality | LC50 | 80000 |
| Labeo rohita | Mortality | LC50 | 10910 |
| Labeo rohita | Mortality | LC50 | 156000 |
| Lemna minor | Population | EC50 | 290 |
| Lemna minor | Population | EC50 | 330 |
| Lemna minor | Population | EC50 | 3140 |
| Lemna minor | Behavior | EC50 | 3270 |
| Lemna minor | Population | EC50 | 5450 |
| Lemna minor | Population | EC50 | 5600 |
| Lemna minor | Population | EC50 | 9420 |
| Lemna minor | Growth | EC50 | 10000 |
| Lemna trisulca | Growth | EC50 | 326.9 |
| Lemna trisulca | Growth | EC50 | 915.3 |
| Lepomis macrochirus | Mortality | LC50 | 3500 |
| Lepomis macrochirus | Mortality | LC50 | 4200 |
| Lepomis macrochirus | Mortality | LC50 | 12500 |
| Lepomis macrochirus | Mortality | LC50 | 12900 |
| Navicula seminulum | Population | EC50 | 1180 |
| Navicula seminulum | Population | EC50 | 1460 |
| Navicula seminulum | Population | EC50 | 1570 |
| Navicula seminulum | Population | EC50 | 1620 |
| Navicula seminulum | Population | EC50 | 1630 |
| Navicula seminulum | Population | EC50 | 2980 |
| Navicula seminulum | Population | EC50 | 3000 |
| Navicula seminulum | Population | EC50 | 3460 |
| Navicula seminulum | Population | EC50 | 3970 |
| Navicula seminulum | Population | EC50 | 4130 |
| Navicula seminulum | Population | EC50 | 4240 |
| Navicula seminulum | Population | EC50 | 4350 |
| Oreochromis mossambicus | Mortality | LC50 | 16500 |
| Oreochromis mossambicus | Mortality | LC50 | 24300 |
| Oreochromis niloticus | Mortality | LC50 | 5906 |
| Oreochromis niloticus | Mortality | LC50 | 65550 |
| Oreochromis niloticus | Mortality | LC50 | 74760 |
| Oreochromis niloticus | Mortality | LC50 | 86410 |
| Selenastrum capricornutum | Population | EC50 | 15 |

**Table S12**

The concentration of heavy metals at each sampling point during the rainy season

| Sampling point | Cd | | Cr | | Cu | | Fe | | Hg | | Mn | | Ni | | Pb | | Zn | |
| --- | --- | --- | --- | --- | --- | --- | --- | --- | --- | --- | --- | --- | --- | --- | --- | --- | --- | --- |
|  | Mean | SD | Mean | SD | Mean | SD | Mean | SD | Mean | SD | Mean | SD | Mean | SD | Mean | SD | Mean | SD |
| SP1 | <LOQ | - | 4.83 | 1.5 | 2.49 | 0.36 | 910.90 | 111.96 | <LOQ | - | 124.21 | 39.05 | 1.26 | 0.20 | <LOQ | - | 17.10 | 1.83 |
| SP2 | <LOQ | - | 3.55 | 0.3 | 2.25 | 0.25 | 707.13 | 41.80 | <LOQ | - | 134.35 | 7.71 | <LOQ | - | 3.23 | 1.14 | 12.77 | 7.34 |
| SP3 | <LOQ | - | 3.98 | 2.1 | 2.37 | 0.45 | 1045.30 | 531.47 | <LOQ | - | 129.01 | 16.82 | 1.42 | 0.49 | <LOQ | - | 7.70 | 9.60 |
| SP4 | <LOQ | - | 2.64 | 0.5 | 2.45 | 0.30 | 695.27 | 124.17 | <LOQ | - | 119.09 | 5.82 | <LOQ | - | <LOQ | - | 8.87 | 3.61 |
| SP5 | <LOQ | - | 2.37 | 0.4 | 2.25 | 0.14 | 628.20 | 29.48 | <LOQ | - | 99.39 | 5.79 | <LOQ | - | <LOQ | - | 6.50 | 0.69 |
| SP6 | <LOQ | - | 2.74 | 0.7 | 2.01 | 0.07 | 743.13 | 130.05 | <LOQ | - | 54.91 | 2.39 | <LOQ | - | <LOQ | - | 7.73 | 8.18 |
| SP7 | <LOQ | - | 2.21 | 0.9 | 2.05 | 0.12 | 581.33 | 76.55 | <LOQ | - | 79.47 | 7.76 | <LOQ | - | <LOQ | - | 5.33 | 2.71 |
| SP8 | <LOQ | - | 1.83 | 0.4 | 2.05 | 0.00 | 480.27 | 88.56 | <LOQ | - | 79.53 | 13.67 | <LOQ | - | <LOQ | - | 12.80 | 8.23 |
| SP9 | <LOQ | - | 1.30 | 0.3 | 2.25 | 0.18 | 402.67 | 40.38 | <LOQ | - | 147.07 | 2.64 | <LOQ | - | <LOQ | - | 9.27 | 5.85 |
| SP10 | <LOQ | - | 2.10 | 0.7 | 1.81 | 0.12 | 386.77 | 146.60 | <LOQ | - | 73.45 | 2.55 | <LOQ | - | <LOQ | - | 6.90 | 1.20 |
| SP11 | <LOQ | - | 1.46 | 0.4 | 2.21 | 0.14 | 206.83 | 22.30 | <LOQ | - | 54.50 | 8.15 | <LOQ | - | <LOQ | - | 7.70 | 1.83 |
| SP12 | <LOQ | - | 1.30 | 0.2 | 2.01 | 0.07 | 203.17 | 21.63 | <LOQ | - | 85.34 | 3.03 | 1.06 | 0.48 | <LOQ | - | 7.70 | 0.69 |
| SP13 | <LOQ | - | 0.98 | 0.2 | 2.09 | 0.30 | 191.60 | 27.58 | <LOQ | - | 62.79 | 3.65 | 1.73 | 0.20 | <LOQ | - | 14.77 | 16.75 |
| SP14 | <LOQ | - | 1.51 | 0.5 | 1.81 | 0.00 | 154.63 | 20.32 | <LOQ | - | 46.58 | 2.65 | 1.38 | 0.41 | <LOQ | - | 9.27 | 7.15 |
| SP15 | <LOQ | - | 2.05 | 0.3 | 2.05 | 0.21 | 412.23 | 52.49 | <LOQ | - | 227.78 | 1.53 | 1.04 | 0.38 | <LOQ | - | 12.40 | 4.90 |
| SP16 | <LOQ | - | 13.51 | 2.1 | 5.63 | 0.21 | 1321.07 | 162.20 | <LOQ | - | 1018.07 | 122.03 | 1.46 | 0.18 | 8.63 | 0.83 | 13.63 | 11.82 |

**Table S13**

The concentration of heavy metals at each sampling point during the dry season

| Sampling point | Cd | | Cr | | Cu | | Fe | | Hg | | Mn | | Ni | | Pb | | Zn | |
| --- | --- | --- | --- | --- | --- | --- | --- | --- | --- | --- | --- | --- | --- | --- | --- | --- | --- | --- |
|  | Mean | SD | Mean | SD | Mean | SD | Mean | SD | Mean | SD | Mean | SD | Mean | SD | Mean | SD | Mean | SD |
| SP1 | <LOQ | - | 0.96 | 0.21 | <LOQ | - | 652.70 | 42.93 | <LOQ | - | 114.78 | 2.30 | <LOQ | - | <LOQ | - | 5.73 | 2.35 |
| SP2 | <LOQ | - | 0.72 | 0.39 | <LOQ | - | 795.57 | 85.56 | <LOQ | - | 124.80 | 0.86 | <LOQ | - | <LOQ | - | 6.50 | 1.91 |
| SP3 | <LOQ | - | 0.55 | 0.06 | <LOQ | - | 705.83 | 65.89 | <LOQ | - | 135.35 | 2.88 | <LOQ | - | <LOQ | - | 2.73 | 0.64 |
| SP4 | <LOQ | - | 1.15 | 0.06 | <LOQ | - | 1129.73 | 89.51 | <LOQ | - | 122.10 | 3.90 | <LOQ | - | <LOQ | - | 3.87 | 1.33 |
| SP5 | <LOQ | - | 0.95 | 0.07 | <LOQ | - | 1050.63 | 125.73 | <LOQ | - | 99.79 | 2.02 | <LOQ | - | <LOQ | - | 7.23 | 1.27 |
| SP6 | <LOQ | - | 0.74 | 0.07 | <LOQ | - | 772.43 | 102.65 | <LOQ | - | 93.85 | 3.33 | <LOQ | - | <LOQ | - | 3.50 | 2.60 |
| SP7 | <LOQ | - | 1.12 | 0.23 | <LOQ | - | 791.70 | 15.32 | <LOQ | - | 143.39 | 0.62 | <LOQ | - | <LOQ | - | 5.73 | 2.81 |
| SP8 | <LOQ | - | <LOQ | - | <LOQ | - | 768.13 | 85.66 | <LOQ | - | 119.30 | 5.25 | <LOQ | - | <LOQ | - | 3.50 | 1.73 |
| SP9 | <LOQ | - | <LOQ | - | <LOQ | - | 1379.33 | 274.29 | <LOQ | - | 112.00 | 4.09 | <LOQ | - | <LOQ | - | 4.67 | 0.64 |
| SP10 | <LOQ | - | <LOQ | - | <LOQ | - | 107.17 | 20.23 | <LOQ | - | 103.91 | 5.18 | <LOQ | - | <LOQ | - | 5.40 | 1.91 |
| SP11 | <LOQ | - | <LOQ | - | <LOQ | - | 575.50 | 40.37 | <LOQ | - | 102.28 | 2.02 | 1.11 | 0.10 | <LOQ | - | 5.73 | 2.81 |
| SP12 | <LOQ | - | <LOQ | - | <LOQ | - | 1020.33 | 66.28 | <LOQ | - | 69.69 | 2.46 | 1.41 | 0.04 | <LOQ | - | 2.40 | 1.73 |
| SP13 | <LOQ | - | <LOQ | - | <LOQ | - | 195.53 | 92.60 | <LOQ | - | 53.91 | 6.26 | 1.53 | 0.02 | <LOQ | - | 6.47 | 2.97 |
| SP14 | <LOQ | - | <LOQ | - | 1.75 | 0.15 | 1013.90 | 106.66 | <LOQ | - | 192.79 | 2.78 | 1.92 | 0.04 | <LOQ | - | 6.87 | 1.68 |
| SP15 | <LOQ | - | 2.67 | 0.15 | 5.62 | 0.69 | 3527.67 | 831.19 | <LOQ | - | 486.67 | 34.27 | 3.22 | 0.09 | <LOQ | - | 19.50 | 2.82 |
| SP16 | <LOQ | - | 15.47 | 0.41 | 3.76 | 0.82 | 5444.33 | 1205.95 | <LOQ | - | 350.43 | 19.54 | 2.11 | 0.45 | <LOQ | - | 9.07 | 1.27 |

**Table S14**

The Water Quality Index (WQI) for each sampling point along the Chao Phraya River.

| Sampling point | Rainy season | | Dry season | |
| --- | --- | --- | --- | --- |
|  | WQI | Water quality status | WQI | Water quality status |
| SP1 | 63.52 | Poor | 67.23 | Poor |
| SP2 | 61.80 | Poor | 60.37 | Poor |
| SP3 | 64.28 | Poor | 54.18 | Poor |
| SP4 | 61.14 | Poor | 60.16 | Poor |
| SP5 | 60.15 | Poor | 58.22 | Poor |
| SP6 | 62.05 | Poor | 57.79 | Poor |
| SP7 | 50.96 | Poor | 58.49 | Poor |
| SP8 | 52.51 | Poor | 54.37 | Poor |
| SP9 | 52.49 | Poor | 59.29 | Poor |
| SP10 | 57.22 | Poor | 62.18 | Poor |
| SP11 | 67.07 | Poor | 79.29 | Very poor |
| SP12 | 74.79 | Poor | 90.67 | Very poor |
| SP13 | 84.27 | Very poor | 87.79 | Very poor |
| SP14 | 86.95 | Very poor | 91.35 | Very poor |
| SP15 | 95.76 | Very poor | 93.22 | Very poor |
| SP16 | 117.14 | Unsuitable for consumption | 121.42 | Unsuitable for consumption |

**Table S15**

The normality test by Shapiro-Wilk results for various water quality parameters

| Variable\Test | Shapiro-Wilk |
| --- | --- |
| (DO) | 0.0006 |
| (pH) | 0.3502 |
| (Conductivity) | < 0.0001 |
| (Cr) | < 0.0001 |
| (Cu) | < 0.0001 |
| (Fe) | < 0.0001 |
| (Mn) | < 0.0001 |
| (Ni) | < 0.0001 |
| (Pb) | < 0.0001 |
| (Zn) | 0.0112 |


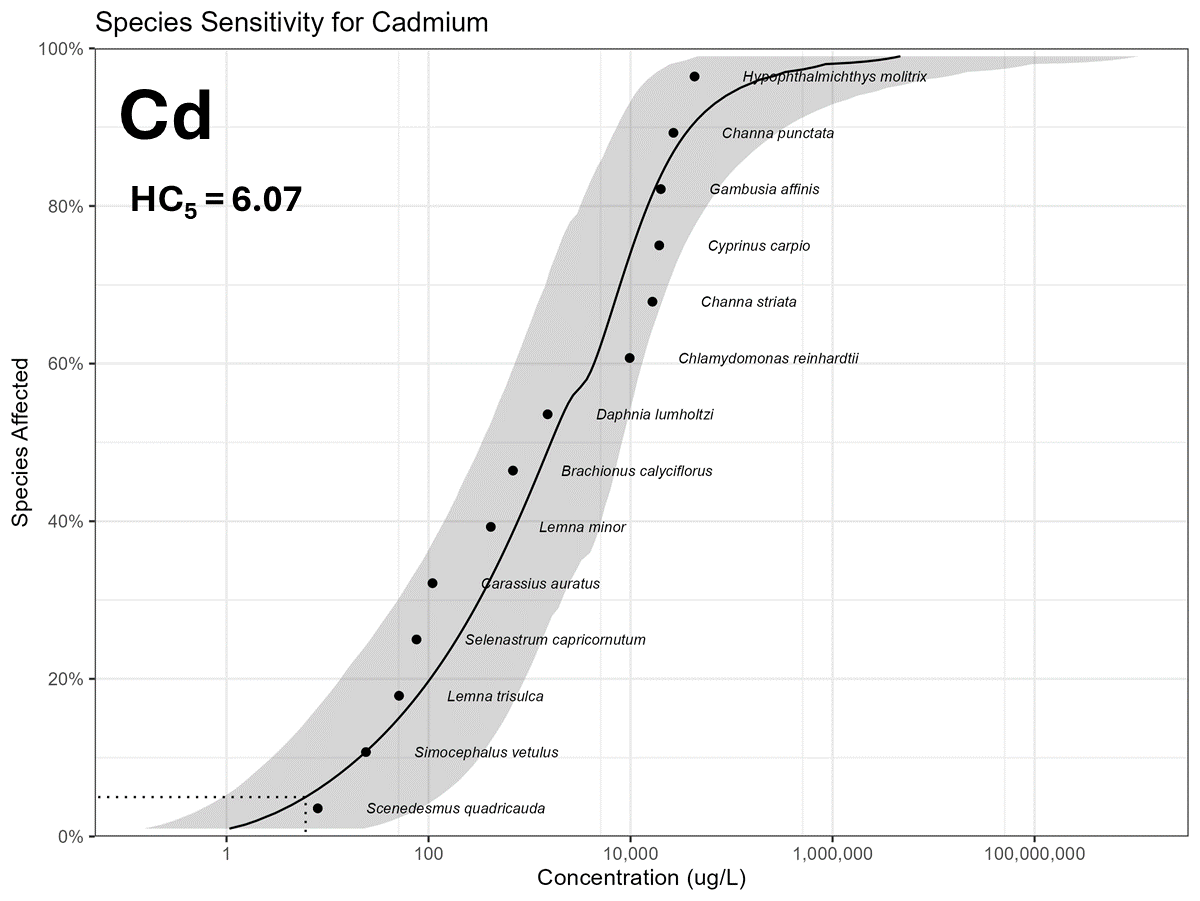


**Fig S1** Species Sensitivity Distributions (SSD) curve of aquatic organisms for Cd


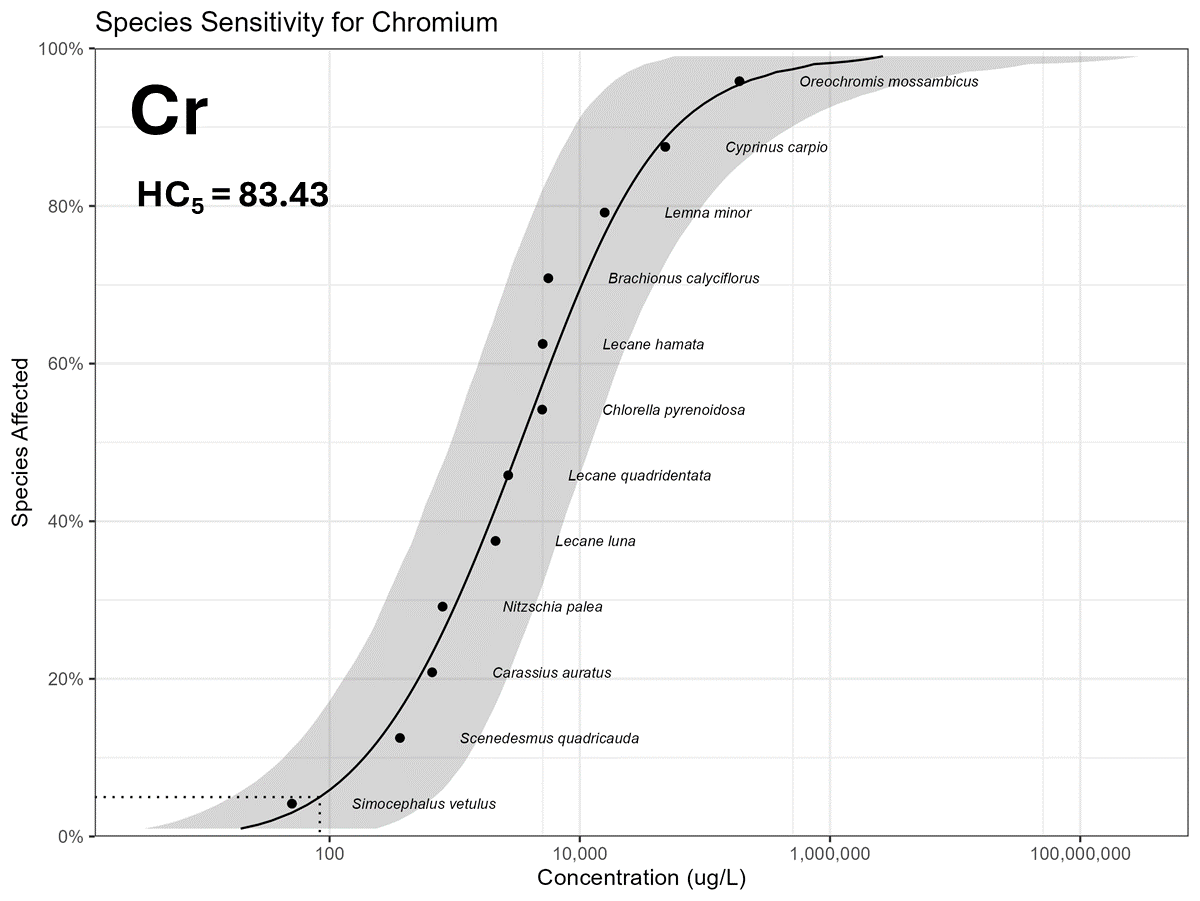


**Fig S2** Species Sensitivity Distributions (SSD) curve of aquatic organisms for Cr

**
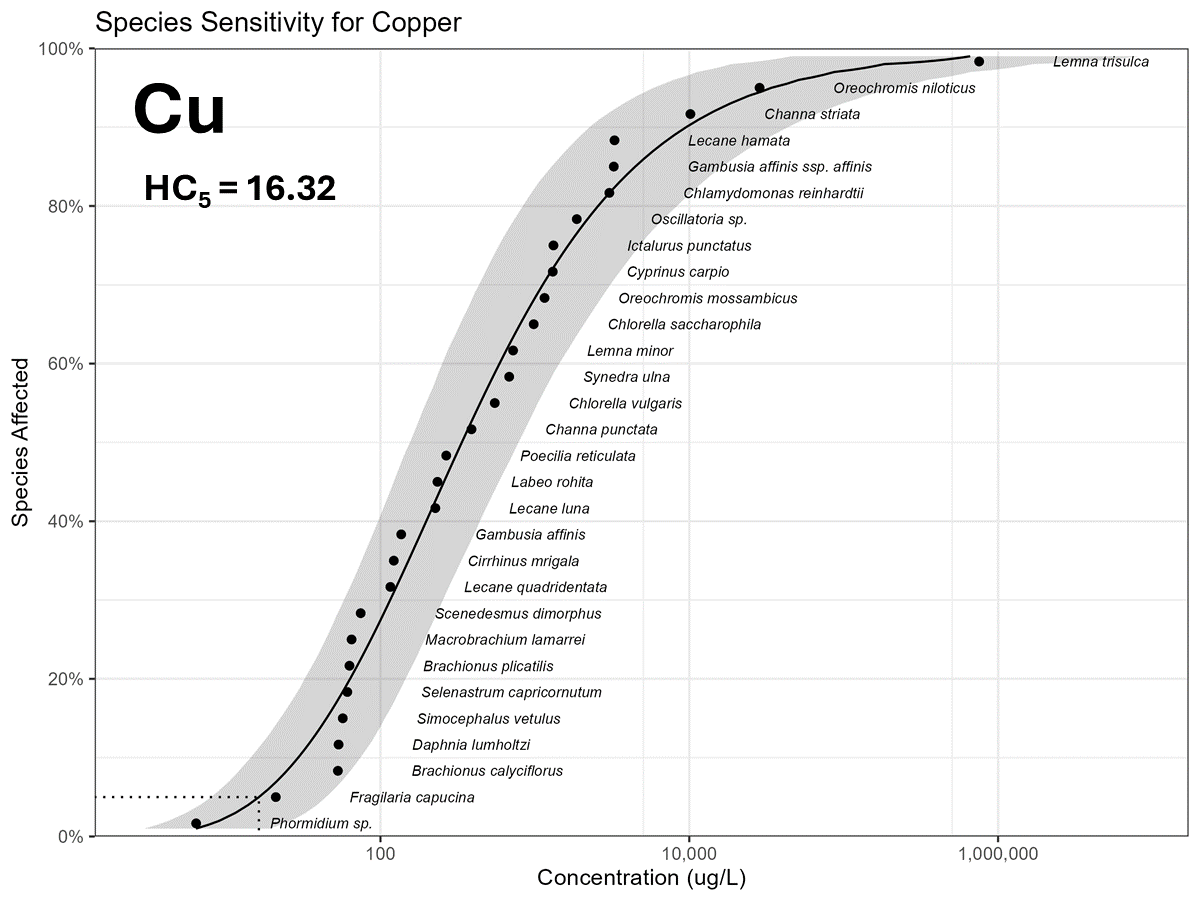
**

**Fig S3** Species Sensitivity Distributions (SSD) curve of aquatic organisms for Cu

**
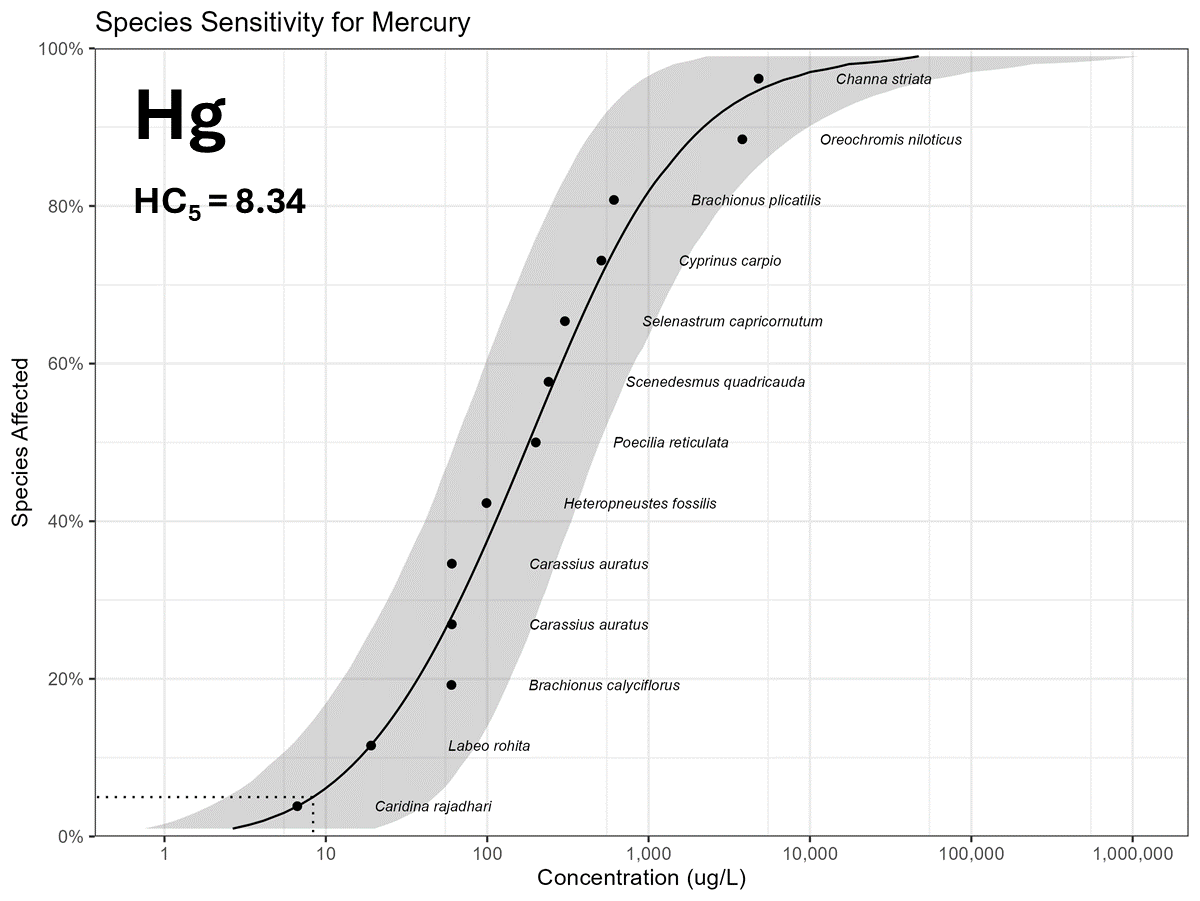
**

**Fig S4** Species Sensitivity Distributions (SSD) curve of aquatic organisms for Hg

**
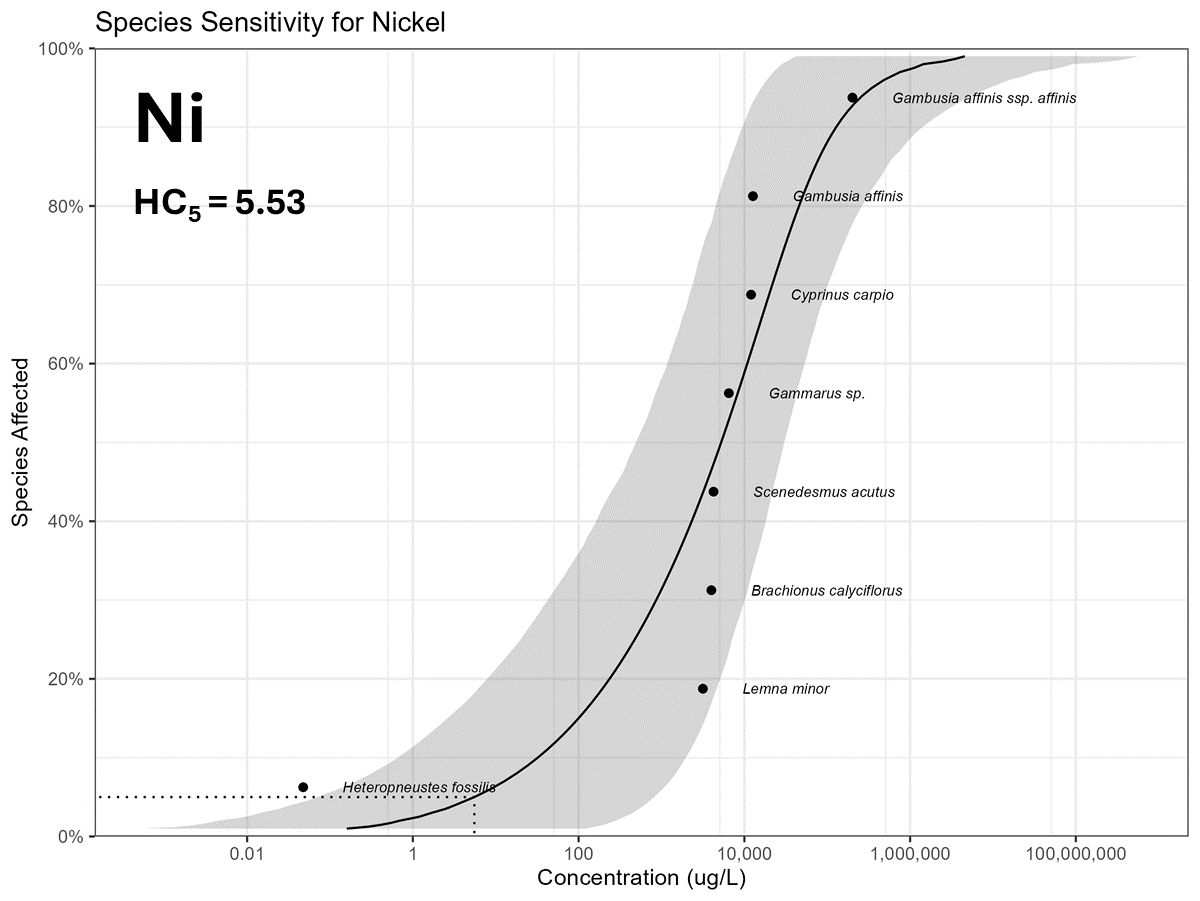
**

**Fig S5** Species Sensitivity Distributions (SSD) curve of aquatic organisms for Ni

**
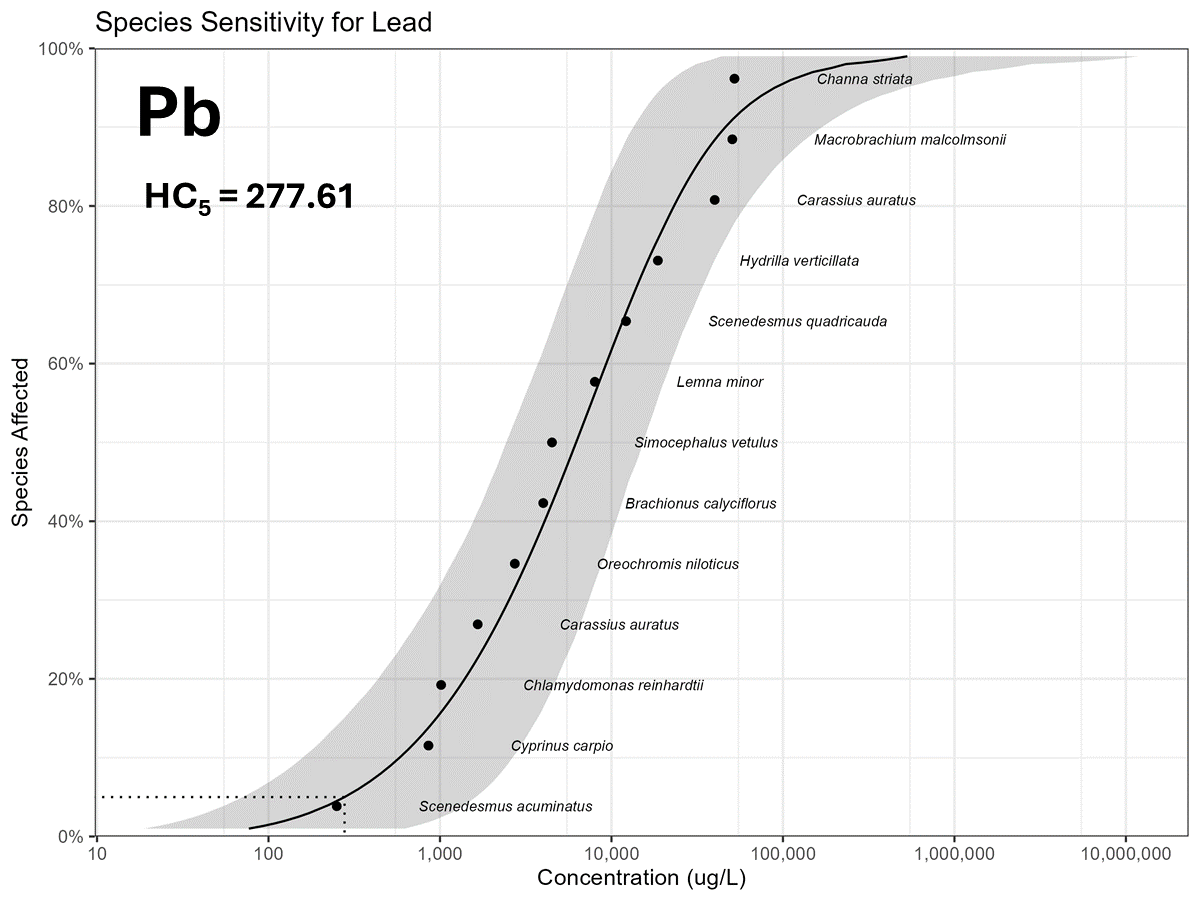
**

**Fig S6** Species Sensitivity Distributions (SSD) curve of aquatic organisms for Pb

**
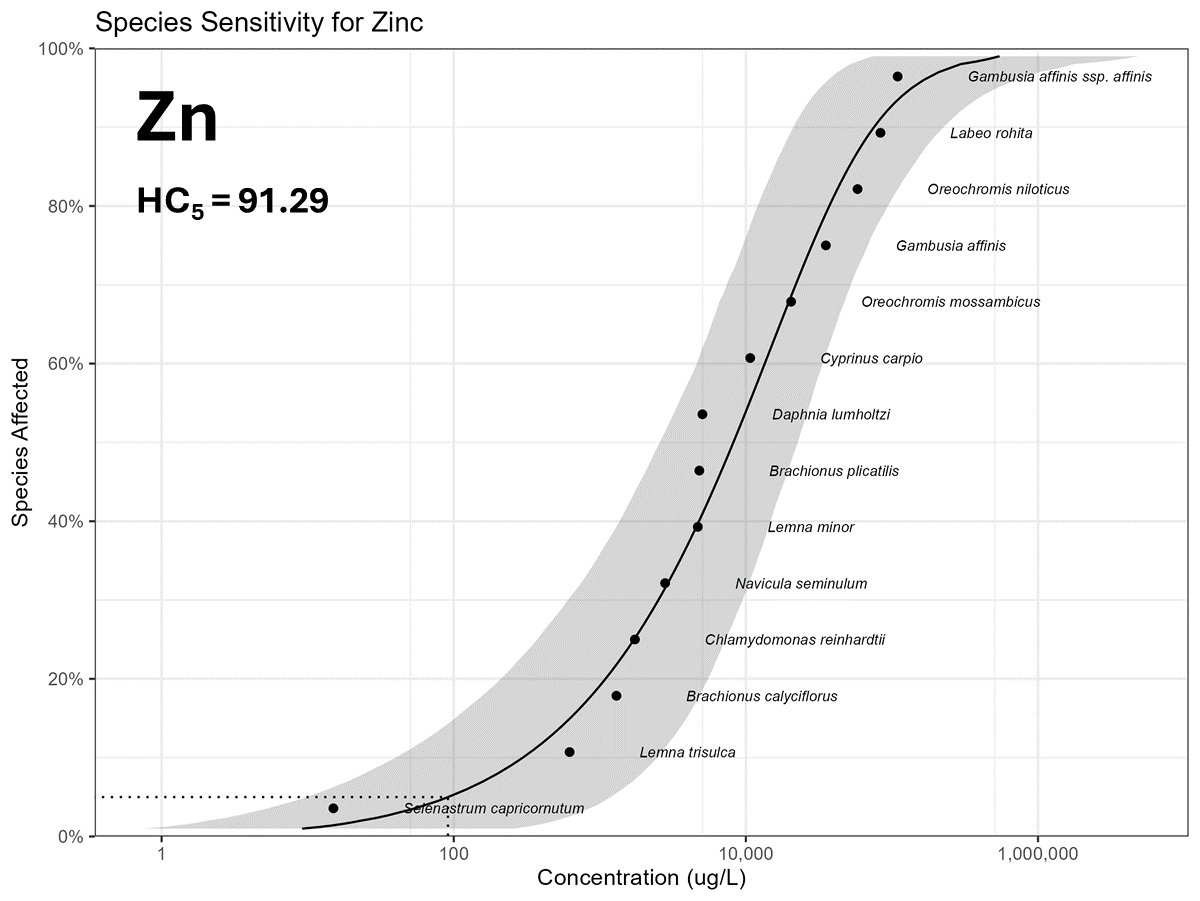
**

**Fig S7** Species Sensitivity Distributions (SSD) curve of aquatic organisms for
